# Maternal age effects on myometrial expression of contractile proteins, uterine gene expression, and contractile activity during labor in the rat

**DOI:** 10.14814/phy2.12305

**Published:** 2015-04-15

**Authors:** Matthew Elmes, Alexandra Szyszka, Caroline Pauliat, Bethan Clifford, Zoe Daniel, Zhangrui Cheng, Claire Wathes, Sarah McMullen

**Affiliations:** 1 Division of Nutritional Sciences, University of NottinghamLoughborough, UK; 2 Royal Veterinary College, Reproduction and Development GroupHatfield, UK

**Keywords:** Maternal age, myometrium, parturition

## Abstract

Advanced maternal age of first time pregnant mothers is associated with prolonged and dysfunctional labor and significant risk of emergency cesarean section. We investigated the influence of maternal age on myometrial contractility, expression of contractile associated proteins (CAPs), and global gene expression in the parturient uterus. Female Wistar rats either 8 (YOUNG *n* = 10) or 24 (OLDER *n* = 10) weeks old were fed laboratory chow, mated, and killed during parturition. Myometrial strips were dissected to determine contractile activity, cholesterol (CHOL) and triglycerides (TAG) content, protein expression of connexin-43 (GJA1), prostaglandin-endoperoxide synthase 2 (PTGS2), and caveolin 1 (CAV-1). Maternal plasma concentrations of prostaglandins PGE_2_, PGF_2*α*_, and progesterone were determined by RIA. Global gene expression in uterine samples was compared using Affymetrix Genechip Gene 2.0 ST arrays and Ingenuity Pathway analysis (IPA). Spontaneous contractility in myometrium exhibited by YOUNG rats was threefold greater than OLDER animals (*P* < 0.027) but maternal age had no significant effect on myometrial CAP expression, lipid profiles, or pregnancy-related hormones. OLDER myometrium increased contractile activity in response to PGF_2*α*,_ phenylephrine, and carbachol, a response absent in YOUNG rats (all *P* < 0.002). Microarray analysis identified that maternal age affected expression of genes related to immune and inflammatory responses, lipid transport and metabolism, steroid metabolism, tissue remodeling, and smooth muscle contraction. In conclusion YOUNG laboring rat myometrium seems primed to contract maximally, whereas activity is blunted in OLDER animals and requires stimulation to meet contractile potential. Further work investigating maternal age effects on myometrial function is required with focus on lipid metabolism and inflammatory pathways.

## Introduction

Delayed child bearing age has been increasing steadily over the last 3 decades within the developed world (Bréart et al. 2003; Ventura et al. 2009). Between the years 1980–2004, the proportion of first births has increased threefold in women aged ≥30, sixfold in women aged ≥35, and 15-fold higher in women aged ≥40 years of age (Martin et al. 2006). This large shift has been attributed to pursuance of professional careers, delaying marriage, and increased availability and widespread use of fertility enhancing therapy. Advancing maternal age is associated with increased risk of complications and adverse outcomes during pregnancy. The greatest risks quantified by Luke and Brown (2007) were prolonged and dysfunctional labor and significant increase in the risk of cesarean section (Kozinszky et al. 2002; Montan 2007). In 2007 the total cesarean delivery rates in the USA were 32% of all births, which had climbed by more than 50% over the previous 10 years (Hamilton et al. 2009) and coincided with the trend of increasing average maternal age at time of first birth (Smith et al. 2008). Cesarean section rates have been reported to be in the range 25–35% for women aged ≥35 years, and 40% in women aged ≥40 years of age, which is significantly higher than the estimated 14% of women younger than 35 years of age that require a cesarean section (Bell et al. 2001).

Although the relationship between maternal age and cesarean section risk is well established, a high reliance on cesarean sections remains a major public health concern (Guihard and Blondel 2001; Joseph et al. 2003a) and the causal mechanism is yet to be fully elucidated. Myometrial biopsies from women of varying parity have been studied *in vitro*, and have provided evidence that advancing maternal age is associated with a reduced degree of spontaneous contraction and contractile strength and an increased likelihood of multiphasic spontaneous myometrial contractions (Smith et al. 2008; Arrowsmith et al. 2012). This association between maternal age and coordination of myometrial contraction indicates adverse effects of aging on control of myometrial activity.

Uterine contractile activity is regulated by the key contractile associated proteins CAV-1, GJA1, and PTGS2. CAV-1 is the structural component of caveolae (Okamoto et al. 1998), omega-shaped invaginations of cell membranes that regulate intracellular signals (Schlegel et al. 1998; Shaul and Anderson 1998). Three different isoforms exist (CAV-1-3) (Okamoto et al. 1998), however, CAV-1 has tight control of contractile transduction pathways as Cav-1 knockout mice exhibit impaired smooth muscle vascular relaxation (Drab et al. 2001). GJA1 is a major myometrial gap junction that facilitates intracellular propagation of electrical impulses (Willecke et al. 2002) and synchronized myometrial contractions. GJA1 plays a key role in parturition, as myometrial loss causes defects in physiological coordination of uterine contractions and prolonged labor(Cluff et al. 2006; Doring et al. 2006). PTGS2 is responsible for regulating uterine activity during pregnancy and parturition through the synthesis of the prostaglandins PGF_2*α*_ and PGE_2_ (Zuo et al. 1994). PGE_2_ causes uterine relaxation and cervical dilation (Lopez Bernal et al. 1993) and is often used in clinical practice to help induce labor(Keirse and de Koning Gans 1995), whereas PGF_2*α*_ stimulates the myometrial contractions that expel the fetus during labor(Challis et al. 1997). Selective inhibition of PTGS2 decreases prostaglandin production and delays induced labor in sheep (Scott et al. 2001) and mice (Gross et al. 2000) and PTGS inhibitors have been used clinically to prevent premature birth (King et al. 2005).

To our knowledge, nobody has yet investigated whether increasing maternal age downregulates expression of the key contractile associated proteins or other genes within the myometrium during labor which may result in reduced myometrial contractile activity. This omission is surprising as age-related changes in gene expression have been reported in rat aortic (Schutzer et al. 2005), colonic (Somara et al. 2007) and penile smooth muscle cells (Bakircioglu et al. 2001) resulting in functional alteration. The release of prostaglandin PGF_2*α*_ from human myometrium obtained at hysterectomy during the secretory phase of the menstrual cycle was also significantly higher in younger women (Quaas et al. 1985).

As current knowledge on the mechanism behind increased risk of cesarean delivery with advanced maternal age is lacking, the purpose of this study was to utilize a rat model to test the hypothesis that modest advances in maternal age alter the pathways leading to activation of myometrial contractile activity during normal term labor. A comparison was made between animals aged 8 weeks (beginning of adolescence) and 24 weeks (mature adults): these ages approximate to 18 and 25 +  years for women (Sengupta 2013).

## Materials and Methods

### Animals and experimental design

All animal work was performed in accordance with the Home Office Guidance on the Operation of the Animals (Scientific procedures) Act 1986. Within the animal facilities at the University of Nottingham, 20 virgin Wistar rat dams (Harlan Ltd., Belton, Leics., UK) either 8 weeks (YOUNG *n* = 10) or 24 weeks of age (OLDER *n* = 10) were fed standard laboratory chow (B&K Universal Ltd., Hull, UK). Rats aged 8 weeks of age have just reached puberty whereas those aged 24 weeks have reached full maturity. Rat dams were then mated naturally with Wistar stud males, and pregnancy confirmed through the appearance of a semen plug on the cage floor. The pregnant rats were then housed individually and maintained on their chow diet throughout gestation until parturition and the birth of the first pup at 22 days gestation. Daily food intake and weight gains were recorded prior to and during pregnancy. At gestational day 20, hourly checks were made for signs of parturition, and following the birth of the first pup, each rat dam was immediately killed by CO_2_ asphyxia and cervical dislocation. Maternal blood samples were collected by cardiac puncture and transferred to heparin tubes, centrifuged at 13,000 g at 4°C for 10 min and the plasma retained for analysis of TAG, cholesterol, prostaglandins PGE_2_, PGF_2*α*,_ and progesterone. The uterus was dissected, fetuses removed and separated from their fetal membrane and placentas and killed by destruction of the brain and decapitation. The uterus was immediately split into two horns, one horn was snap frozen and stored at −80°C until subsequent analysis of TAG and cholesterol content and expression of the contractile associated proteins, GJA1, CAV-1, and PTGS2 or used for RNA isolation and microarray analysis. The second horn was immediately stored at 4°C in modified Krebs–Henseleit buffer that had been gassed with 95% 0_2_ and 5% CO_2_ (NaCl, 119 mmol/L; KCl, 4.69 mmol/L; MgSO_4_, 1.17 mmol/L; KH_2_PO_4_, 1.18 mmol/L; NaHCO_3_, 25 mmol/L; Glucose, 5.5 mmol/L; and CaCl_2_, 2.5 mmol/L, adjusted to pH 7.4) and used for myometrial contractile studies within 12 h.

### Myometrial contractile analysis

Small strips (1 × 5 mm) of longitudinal myometrium were dissected from the uterine horn from each animal and each tissue strip was suspended in a separate 25 mL organ bath (Letica, AD Instruments, Oxford, UK) filled with modified Krebs–Henseleit buffer (detailed above) maintained at 37°C, and gassed with 95% 0_2_ and 5% CO_2_. Myometrial strips were then secured with cotton and placed under isometric conditions with a 20 mN resting tension. Contractile activity for each myometrial tissue strip was recorded using isometric force transducers connected to a bridge amplifier, which was in turn connected to a dedicated data acquisition system (Powerlab/8SP, AD Instruments, Oxford, UK) and recorded and analyzed by Chart software (version 7; PowerLab, AD Instruments). Myometrial strips were then left to stabilize for 30 min until regular phasic contractions were achieved. Following the equilibration period and the generation of stable, reproducible contractions, 30 min baseline spontaneous contractile function was then determined before the cumulative addition of either PGF_2*α*_ (10^−10^ to 10^−6^ mol/L), phenylephrine (10^−10^ to 10^−3^ mol/L), or carbachol (10^−10^ to 10^−3^ mol/L) applied at 10-min intervals (all from Sigma-Aldrich, Poole, Dorset, UK). The resultant contractile activity measured during baseline and each 10 min drug accumulation included activity integrals (area under the time-force curve), peak force (maximum tension above basal force), and frequency of contractions. Viability of myometrial strips was checked at the end of each experiment by addition of KPSS (modified Krebs–Henseleit solution as detailed above with equimolar replacement of sodium with 20 mmol/L of potassium).

### Total cholesterol and triglyceride assays

Lipids were extracted from 300 mg of uterine tissue by homogenizing in a mixture of hexane/isopropanol (3:2 v/v) for 5 mins. The contents were then centrifuged at 2000 g for 5 mins at 25°C. The resulting liquid phase was carefully removed and dried under liquid nitrogen for 1 h. The dried extract was then dissolved in 1 mL isopropanol and analyzed. Total cholesterol and triglycerides in the maternal plasma and uterine tissue were assayed through a commercial kit (ThermoTrace, Noble Park, Vic., Aus) according to the manufacturers’ instructions. Standard curves ranging from 0–5 mmol/L and 0–3.5 mmol/L were produced for cholesterol and triglycerides, respectively. On a 96 well plate 200 *μ*L of cholesterol or triglyceride assay reagent was added to 10 *μ*L of sample or standard, and incubated for 15 mins at 37°C. The absorbance was then read at 550 nm (with a reference wave length of 655 nm).

### Western blot analysis

For analysis of uterine expression of GJA1, CAV-1, and PTGS2, one frozen uterine horn was ground to a powder in liquid nitrogen and homogenized briefly for 30 sec in ice cold buffer containing 5 mmol/L Tris pH 7.4, 2 mmol/L EDTA and protease inhibitor cocktail (Calbiochem, San Diego, CA, USA). Homogenates were then split into three parts for analysis of each protein. Homogenate for PTGS2 underwent centrifugation at 13,000 g and both GJA1 and CAV-1 was spun at 3500 g for 15 min at 4°C and the supernatants extracted. Protein concentrations of each supernatant were determined using the Bio-Rad protein assay system (Bio-Rad, Hemel Hempstead, UK) according to the manufacturer's instructions. Samples were standardized to a concentration of 4 mg/mL with Laemmli's sample buffer (62.5 mmol/L Tris pH6.8, 2% SDS, 10% glycerol, 0.02% bromophenol blue, 150 mmol/L dithiothreitol) and boiled for 3 min before equal protein quantities of each sample were separated by SDS PAGE. Proteins were transferred to nitrocellulose membrane (Hybond-C extra, Amersham Bioscience) for probing with primary antibodies to (1) PTGS2 (Santa Cruz Biotechnology Inc; rabbit polyclonal raised against amino acids 50-111 of PTGS2 of human origin), (2) GJA1 (Cell Signaling; rabbit polyclonal against a synthetic peptide corresponding to residues of human GJA1 and (3) CAV-1 (Cell Signaling; rabbit monoclonal against a synthetic peptide corresponding to residues near the amino terminus of human CAV-1). Membranes were incubated in blocking solution (5% dried skimmed milk in TBS with 1% Tween 20) prior to incubation with primary antibodies. Horseradish peroxidise secondary antibody conjugated to rabbit IgG was used at a working concentration of 1:5000 (GE Healthcare, Amersham, UK). Bands were developed on high-performance chemiluminescence film (Hyperfilm ECL, Amersham) using ECL reagent (GE Healthcare). Densitometric analysis of band intensity was performed using a Biorad Gel Doc XR imaging system and Quantity One 1D analysis software.

### Radioimmunoassay for PGF_2α_, PGE_2_, and progesterone

The concentration of PGF_2*α*,_ PGE_2_, and progesterone in maternal plasma was quantified using established radioimmunoassays (Wathes et al. 1986; Leung et al. 2001). The tritiated tracers ([5, 6, 8, 9, 11, 12, 14, 15 (*n*)-^3^H]- PGF_2*α*_, 6,8,9,11,12,14,15 (*n*)-[^3^H] PGE_2_ and [1,2,6,7,16,17-^3^H] progesterone), were from PerkinElmer (Cambridge, UK) and standards were supplied by Sigma-Aldrich. The antisera against prostaglandins PGF_2*α*,_ and PGE_2_ were a kind gift from Dr N.L. Poyser (University of Edinburgh, Edinburgh, UK) and progesterone was from Dr M. Sauer (Veterinary Laboratory Agency, Weybridge, Surrey). The concentrations of PGF_2*α*_, PGE_2_, and progesterone were calculated using a semilogarithmic plot. The limit of detection for PGF_2*α*_ and PGE_2_ was 1 and 2 pg/tube with an intra-assay Co-Var value of 4.1% and 3.5%, respectively. The limit of detection and intra-assay coefficient of variation for progesterone was 16 pg/tube and 6.6%, respectively.

### RNA extraction

Total RNA was extracted from 25 mg of frozen uterine tissue using an RNA isolation kit (Roche, Burgess Hill, UK. High Pure RNA Tissue Kit). To disrupt the tissue and lyse the cells, 400 *μ*L of Lysis/Binding Buffer was added directly to the frozen tissue and homogenized. The resulting lysate was centrifuged for 2 min at 13,000 × g. The supernatant was collected and mixed well with 200 *μ*L of absolute ethanol. The 600 *μ*L sample was then centrifuged through the High Pure Filter Tube spin column at 13,000 × g for 30 sec (at room temperature for all centrifugations). The flow through was discarded after each step. RNA caught on the membrane of the spin column was treated with 10 *μ*L DNase I and 90 *μ*L of DNase-incubation buffer for 15 min at room temperature. 500 *μ*L of wash solutions I and II (containing ethanol to ensure the RNA was precipitated and thus remained in the spin column membrane) were passed through the spin column through centrifugation at 8000 × g for 15 sec. A further 300 *μ*L of wash solution II was passed through the column via centrifugation at 13,000 × g for 2 min to ensure all contaminants were removed from the RNA on the spin column membrane. Finally, the RNA was eluted in 50 *μ*L of RNase-free H_2_O into a fresh 1.5 mL tube via centrifugation at 8000 × g for 1 min and stored at −80°C. RNA concentration and purity were determined using the NanoDrop ND-1000 spectrophotometer (NanoDrop Technologies, Wilmington, DE).

### Microarray analysis

Microarray hybridization and data acquisition were carried out in ARK-Genomics (Roslin Institute, Edinburgh, UK) using Affymetrix Genechip Rat Gene 2.0 ST arrays based on their established protocols. The acquired data were analyzed with GeneSpring GX V12.5 software package (Agilent Technologies, Santa Clara, CA 95051). The probe pairs were summarized into a single value per gene using robust multichip analysis with Quantiles normalization. After filtration and summarization, 29,489 probes/genes were available. The differentially expressed genes were identified using Moderate *t*-test at *P* = 0.05 with Benjamini & Hochberg (BH) false discovery rate adjustment for multiple tests.

### Pathway analysis

The annotated genes were organized using Entrez Gene combined with gene symbols as identifiers and fold changes and adjusted *P* values as observations. They were loaded into Ingenuity Pathway Analysis (IPA) V7.5 software server (Ingenuity, Redwood City, CA) for mapping into relevant functional groups and pathway analysis.

### Quantitative real-time PCR

Total RNA was reverse transcribed using a cDNA synthesis kit (Roche, Transcriptor First Strand cDNA Synthesis Kit) with random primers. RNA (500 ng) was mixed with 2 µL Random Hexamer Primers and water to give a final volume of 13 µL and incubated at 65°C for 10 min. The samples were immediately cooled on ice and to each template-primer mix were added 4 µL Transcriptor Reverse Transcriptase Reaction Buffer, 0.5 µL Protector RNase Inhibitor, 2 µL Deoxynucleotide Mix, and 0.5 µL Transcriptor Reverse Transcripase. A master mix of reagents was prepared for the above reaction to minimize potential variation from pipetting. Selected negative control samples were also prepared by including all reagents as above, minus the reverse transcriptase. The reactions tubes were then incubated at 25°C for 10 min, followed by 55°C for 30 min and the enzyme then inactivated by heating to 85°C for 5 min and the reaction stopped by cooing to 4°C. Assays were designed for 18 genes of interest (see Table S1). Cyclophilin was also analyzed as a housekeeping gene. Primer sequences were designed using Primer Express (Applied Biosystems) based on the target RNA sequence and alignment specificity and compatibility were checked using BLAST (National Center for Biotechnology Information) and primers were purchased from Sigma (UK). Gene symbols, sequence information, and accession numbers are provided in Table S1.

Real-time PCR was conducted on a Lightcycler 480 (Roche, Burgess Hill, UK). Reactions were carried out in triplicate on 384 well plates. Each well contained 5 *μ*L of cDNA with the following reagents: 7.5 *μ*L SYBR green master mix (Roche), 0.45 *μ*L forward, and reverse primers (10 *μ*mol/L each; final concentration 0.3 *μ*mol/L each) and 1.6 *μ*L RNase-free H_2_O (total volume of 15 *μ*L per well). Samples were preincubated at 95°C for 5 min followed by 45 PCR amplification cycles (denaturation: 95°C for 10 sec; annealing: 60°C for 15 sec; elongation: 72°C for 15 sec). A standard curve was produced using serial dilutions of a pool of cDNA made from all samples to check the linearity and efficiency of the PCR reactions. Transcript abundance was determined using the standard curve.

### Statistical analysis

All data apart from the microarray data and pathway analysis were analyzed using the Statistical Package for Social Science (Vers 16; SPSS Inc, Chicago, IL) and expressed as the mean value with standard error, and *P* < 0.05 was considered as statistically significant. The effect of maternal age on measured outcomes was determined through use of one way ANOVA. For dose–response curves each replicate was considered as an individual point, as a result each curve represents the mean of *n* = 4 or 7. The dose–response curves were fitted using the standard least squares (ordinary) fit method. The effects of maternal age on logEC_50_ values of curves for integral contractile activity were analyzed by GraphPad Prism (version5; GraphPad, Inc., San Diego, CA) using a sigmoidal dose–response (variable) slope curve and two-tailed t-test to investigate the null hypothesis that logEC_50_ was the same for each dataset. The sigmoidal dose–response curve (variable slope) is defined by the four parameter logistic equation *y* = bottom + (top–bottom)/(1 + 10^(log EC50−x)Hillslope)^). Array data were analyzed as described above. qPCR data were analyzed by t-test and log transformed if not of equal variance.

## Results

### Maternal weight gain and litter size

As a key component of this study was to look at the effects of maternal age on myometrial contractile activity, we would expect to see significant differences in maternal weight. Post mating, the average weight of YOUNG rats at 8 weeks of age of 196 ± 2 g was significantly lower than the 280 ± 5.9 g observed in the OLDER animals at 24 weeks (*P* < 0.0001). This difference in weight persisted throughout pregnancy such that at gestational day 21 the average weight in YOUNG rats was 314.6 ± 10.4 g compared to 373.9 ± 5.5 g in the OLDER animals (*P* < 0.0001). Although OLDER rats were still significantly heavier than their YOUNG counterparts at the end of pregnancy, the YOUNGER animals gained 118.4 ± 7 g, whereas OLDER animals only gained 93.4 ± 5.2 g. Litter size was not affected by maternal age, but there was a trend for the average pup weight to be raised slightly in the OLDER rats (*P* = 0.055) (see Table1). All dams gave birth on gestational day 22 but it was not possible to determine accurately whether there was a significant difference between timing of labor with maternal age as a number of female rats plugged overnight during mating.

**Table 1:** Summary of the effects of maternal age on the parameters measured in pregnant rats used in the study

Parameter	Young	Older	*P* value
*n* number	6	7 or 8	
Age at mating (weeks)	8	24	
Weight at mating (g)	196 ± 2.0	280 ± 5.9	***P***** < 0.0001**
Weight at delivery (g)	314.6 ± 10.4	373.9 ± 5.5	***P***** < 0.0001**
Weight gain during pregnancy (g)	118.4 ± 7.0	93.4 ± 5.2	***P***** < 0.015**
Litter size	9.4 ± 0.6	9.5 ± 1.2	0.834
Litter weight (g)	51.0 ± 3.0	55.2 ± 5.7	0.749
Average pup weight (g)	5.4 ± 0.15	5.9 ± 0.15	0.055
*Maternal plasma*			
Cholesterol (mmol/L)	2.39 ± 0.25	2.0 ± 0.21	0.178
TAG (mmol/L)	0.9 ± 0.25	1 ± 0.17	0.652
Progesterone (ng/mL)	25.9 ± 1.7	33.9 ± 4.5	0.147
PGF_2α_ (ng/mL)	0.045 ± 0.01	0.065 ± 0.03	0.552
PGE_2_ (ng/mL)	0.22 ± 0.01	0.3 ± 0.02	0.666
*Myometrial tissue*			
Cholesterol (μmol/L/mg)	51.1 ± 7.6	47.9 ± 4.6	0.715
TAG (μmol/L/mg)	52.6 ± 14.6	50.4 ± 10.9	0.905
GJA1 (relative density to β-actin)	41189 ± 9681	42439 ± 6817	0.718
PTGS2 (relative density to β-actin)	14660 ± 3900	11066 ± 2931	0.469
CAV-1 (relative density to β-actin)	16189 ± 3638	14222 ± 2993	0.684

### Effects of maternal age on spontaneous contractile activity

**Figure 1:**
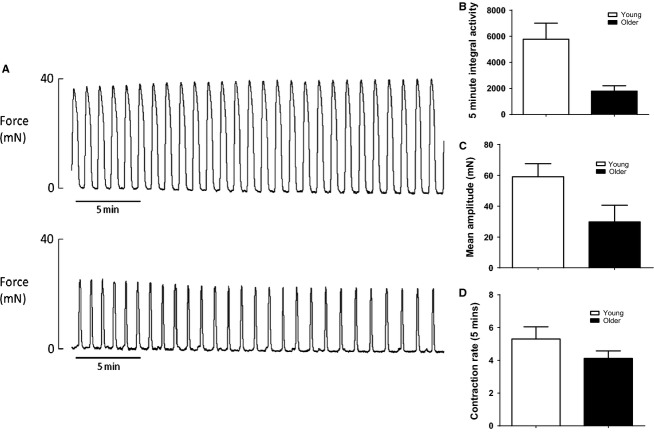
The effects of maternal age on spontaneous uterine contractions in laboring rats. (A) Represents recordings of spontaneous uterine contractile activity in a YOUNG (*top trace*) and OLDER (*bottom trace*) rat dam. B, C, and D are different measures of contractile activity in the myometrium of YOUNG (*n* = 4) and OLDER (*n* = 7) rats where (B) Represents 5 min integral activity, (C) Mean amplitude of contraction, and (D) 5 min contraction rate. Data were analyzed by one way ANOVA and significant differences between maternal age were determined at the *P* < 0.05 level. Statistical analysis revealed that spontaneous integral activity and contraction rate was significantly greater in myometrial strips of YOUNG compared to OLDER rats, with values of *P* < 0.03 and *P* < 0.05, respectively. Although the mean amplitude of spontaneous contractions was also higher in YOUNG animals versus their OLDER counterparts, it did not reach significance with *P* = 0.057.

Myometrial strips were obtained post mortem on day 22 of pregnancy, immediately after birth of the first pup. They were mounted in an organ bath and allowed to start contracting spontaneously. Once they reached stable rhythmic contractions (within approximately 20 min of mounting), a 30 min baseline period of spontaneous contractile activity was recorded to determine mean integral activity, amplitude of contraction, and contractile rate (Fig.1A). It was clear that myometrial tissue from YOUNG laboring rats exhibited greater spontaneous myometrial contractile activity than laboring myometrium from OLDER rats. Evidence to support this is that YOUNG rats had a significantly greater integral activity (*P* < 0.03, Fig.1B) and rate of contraction (*P* < 0.05, Fig.1D) compared to their OLDER counterparts. Similarly, laboring myometrial tissue from YOUNG rats exhibited greater spontaneous contractile strength compared to OLDER rats (Fig.1C), which was just short of reaching significance (*P* = 0.057).

### Effects of maternal age on the contractile response to PGF_2*α*_, phenylephrine, and carbachol

**Figure 2:**
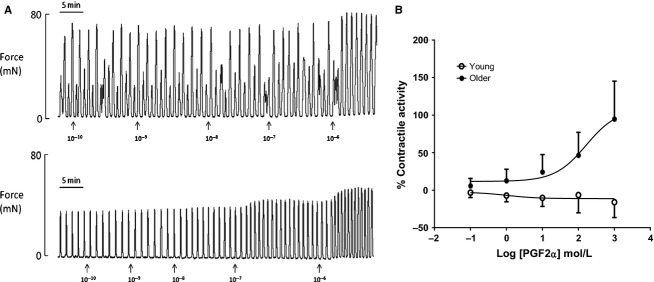
Stimulation of uterine contractile activity with PGF_2*α*_. (A) Representative recordings of stimulated uterine contractions in a YOUNG (*top trace*) and OLDER (*bottom trace*) rat dam by the accumulative addition of PGF_2*α*_ (range 0.1 nmol/L to 1 *μ*mL/L, units shown are in log mol/L). (B) A dose–response curve to determine the effects of maternal age (YOUNG (*n* = 4) or OLDER (*n* = 7) on uterine integral activity to increasing doses of PGF_2*α*_ (range 0.1 nmol/L to 1 *μ*mL/L, units shown are in log mol/L). Statistical analysis reveals that the dose–response curves were significantly different (*P* < 0.0005). LogEC_50_ was significantly shifted by maternal age, YOUNG logEC_50 _= 0.009, OLDER logEC_50_ =2.22.

With evidence suggesting that spontaneous myometrial activity is altered by maternal age it was important to determine whether the myometrial response to known agonists was also affected. To stimulate an increase in myometrial contractile activity, myometrial strips were incubated with increasing doses of PGF_2*α*_ (Fig.2A). The spontaneous contractile activity in myometrial strips from YOUNG laboring rats did not respond as contractile activity did not improve any further with increasing PGF_2*α*_ concentration, suggesting that the YOUNG myometrium was already contracting maximally (Fig.2A *top trace*). In contrast, myometrial strips from OLDER rat dams were sensitive to PGF_2*α*_ stimulation. Contractile activity improved every time PGF_2*α*_ concentration was increased (Fig.2A *bottom trace*). Thus, dose–response curves to PGF_*2α*_ were significantly different between age groups, being more sensitive in OLDER rats (*P* < 0.0005, Fig.2B).

**Figure 3:**
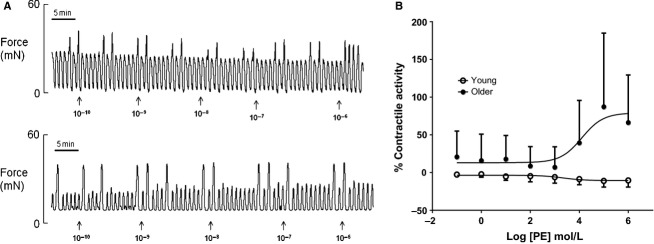
Effects of phenylephrine on uterine activity. (A) Representative recordings of uterine contractions in a YOUNG (*top trace*) and OLDER (*bottom trace*) rat dam incubated with accumulative concentrations of Phenylephrine (range 0.1 nmol/L to 1 *μ*mL/L, units shown are in log mol/L). (B) A dose–response curve to determine the effects of maternal age, YOUNG (*n* = 4) or OLDER (*n* = 7) on uterine integral activity to increasing doses of phenylephrine (range 0.1 nmol/L to 1 mmol/L, units shown are in log mol/L). Statistical analysis reveals that the dose–response curves were significantly different (*P* < 0.0001). LogEC_50_ was significantly shifted by maternal age, YOUNG logEC_50 _= 3.34, OLDER logEC_50_ =4.1.

**Figure 4:**
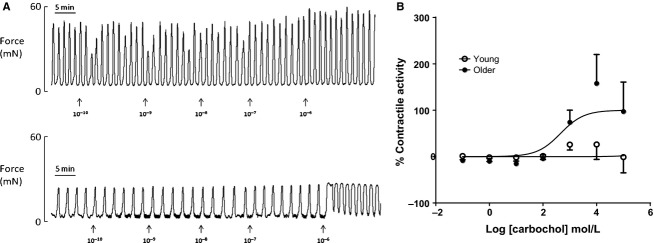
Effects of carbachol on uterine contractile activity. (A) Representative recordings of uterine contractile activity in a YOUNG (*top trace*) and OLDER (*bottom trace*) rat dam incubated with accumulative concentrations of Carbachol (range 0.1 nmol/L to 1 *μ*mL/L, units shown are in log mol/L). (B) A dose–response curve to determine the effects of maternal age, YOUNG (*n* = 4) or OLDER (*n* = 7) on uterine integral activity to increasing doses of Carbachol (range 0.1 nmol/L to 1 mmol/L, units shown are in log mol/L. Statistical analysis reveals that the dose–response curves were significantly different (*P* < 0.0002). LogEC_50_ was significantly shifted by maternal age, YOUNG logEC_50 _= 6.73, OLDER logEC_50 _= 2.66.

Similar findings were observed when myometrial strips were treated with increasing concentrations of phenylephrine and carbachol (Figs.3A, 4A). Myometrial strips from YOUNG animals did not respond to either drug (Figs.3A, 4A, *top traces*) whereas laboring myometrium from OLDER rat dams was more sensitive (*P* < 0.01) to both phenylephrine and carbachol and responded positively by increasing contractile activity with an increase in concentration (Figs.3A, 4A, *bottom traces,* Figs3B, 4B).

### Lipid profiles

With physiological data suggesting that the contractile activity of laboring myometrium was altered with maternal age, it was important to determine the possible mechanism. As both plasma and tissue cholesterol concentrations have been observed to play a key role in smooth muscle contraction (Babiychuk et al. 2004; Smith et al. 2005), we determined the total cholesterol and TAG concentration in the maternal plasma and uterine tissue from YOUNG and OLDER rat dams. The cholesterol and TAG concentrations were not significantly different between 2 and 6 months of maternal age (Table1).

### Uterine expression of contractile associated proteins

Western blot analysis of the key contractile associated proteins GJA1, PTGS2, and CAV-1 in uterine tissue from YOUNG and OLDER rat dams provided evidence that maternal age did not significantly alter the expression of contractile associated proteins during parturition (Table1).

### Maternal plasma concentrations of progesterone, PGF_2α_, and PGE_2_

Although maternal plasma concentrations of progesterone were numerically lower in YOUNGER compared to OLDER rat dams at labor onset (25.9 ± 1.7 ng/mL vs. 33.9 ± 4.5 ng/mL), this difference was not significant due to greater variability in the older animals (Table1). Maternal age had no effect on the circulatory levels of PGF_2*α*_ and PGE_2_ (Table1).

### Array analysis and pathways

To try and unravel the possible mechanism behind altered myometrial contractile activity during labor we next compared global gene expression profiles between the two age groups. Microarray analysis of the uterine horn samples identified that in the 327 genes with real fold change >1.5, 181 were significantly different (*P* < 0.05) between 3 and 6-month-old rats. Of this total, 129 genes were significantly downregulated (71%) and 51 genes were upregulated (29%) in the 6-month-old rats compared with the younger rats (see Table S2). Initial analysis of the top 20 differentially expressed genes (DEG) that were decreased by 2- to 10-fold during labor in the older dams found many that are known to be involved in lipid transport and metabolism (*Afp, Apob, Apoh, Apom, Apoc2, and Olr1*) and immune or inflammatory response (*Ceacam11, Prl, Pramef12, H19, Mmp3, Gzmb, Prf1, Aoc1*) (Table2). The top 20 upregulated genes with 1.5 to 3-fold increases in older animals were also involved in immune and inflammatory responses (including *Scgb1a1, Serpina3n, Cxcl6, Ctse, Cd79a, Noxa1, Ptgs2, and Foxa1*) and signaling through G-protein-coupled receptors (GPCR) and ion channels (including *Fxdy3, Gabrp, Clic6, Sctr, and Inmt*) (Table3). Even though Ptgs2 gene expression was upregulated 1.8-fold, this was not reflected in changes in protein expression of PTGS2 or circulating PGF_2*α*_ and PGE_2_ (Table1).

**Table 2:** Top 20 genes ranked by real fold decrease in uterine horn from 8-week-old laboring rats (YOUNG) compared to 24-week-old laboring rats (OLDER)

Fold decrease	Entrez gene ID	Unigene ID	Gene symbol	Entrez gene name
−10.0	24177	Rn.9174	*Afp*	Alpha-fetoprotein
−8.8	54225	Rn.33815	*Apob*	Apolipoprotein B (including Ag(x) antigen)
−6.9	287774	Rn.1824	*Apoh*	Apolipoprotein H (beta-2-glycoprotein I)
−5.9	*	*	*Prl*	Prolactin family 2–8, subfamily, and members
−4.8	292668	Rn.205326	*Ceacam11*	Carcinoembryonic antigen-related cell adhesion molecule 11
−4.3	24856	Rn.1404	*Ttr*	Transthyretin
−4.2	55939	Rn.262	*Apom*	Apolipoprotein M
−3.9	315907	Rn.214057	*Nrk*	Nik-related kinase
−3.8	25105	Rn.3835	*Nppb*	Natriuretic peptide type B
−3.8	309122	Rn.6171	*H19*	H19, imprinted maternally expressed transcript (nonprotein coding)
−3.7	691157	Rn.182598	*Pramef12*	PRAME family member 12
−3.7	24483	Rn.118681	*Igf2*	Insulin-like growth factor 2 (somatomedin A)
−3.3	25065	Rn.14799	*Slc12a1*	Solute carrier family 12 (sodium/potassium/chloride transporters), member 1
−3.3	292697	Rn.16843	*Apoc2*	Apolipoprotein C-II
−3.1	363493	Rn.46187	*Taf7 l*	TAF7-like RNA polymerase II, TATA-box-binding protein (TBP)-associated factor, 50 kDa
−3.0	171045	Rn.32086	*Mmp3*	Matrix metallopeptidase 3 (stromelysin 1, progelatinase)
−2.9	171528	Rn.21395	*Gzmb*	Granzyme B (granzyme 2, cytotoxic T-lymphocyte-associated serine esterase 1)
−2.9	29366	Rn.2271	*Serpine2*	Serpin peptidase inhibitor, clade E (nexin, plasminogen activator inhibitor type 1), member 2
−2.8	140914	Rn.87449	*Olr1*	Oxidized-low-density lipoprotein (lectin-like) receptor 1
−2.7	50669	Rn.11206	*Prf1*	Perforin 1 (pore-forming protein)
−2.6	65029	Rn.54493	*Aoc1*	Amiloride-binding protein 1 (amine oxidase (copper-containing))

All *P* < 0.05.

* Represented by >1 probe on the array; and include significant fold increases in 10 genes including Prl2a1, 2c1, 4a1, 5a1, 5a2, 6a1, 7a3, 7b1, 7d1, and 8a5.

**Table 3:** Top 20 genes ranked by real fold increase in uterine horn from 8-week-old laboring rats (YOUNG) compared to 24-week-old laboring rats (OLDER)

Fold increase	Entrez gene ID	Unigene ID	Gene symbol	Entrez gene name
5.1	25575	Rn.2206	*Scgb1a1*	Secretoglobin, family 1A, member 1 (uteroglobin)
3.0	116831	Rn.3896	*Fxdy3*	FXYD domain containing ion transport regulator 3
2.6	81658	Rn.21401	*Gabrp*	Gamma-aminobutyric acid (GABA) A receptor, pi
2.2	304081	Rn.214050	*Clic6*	Chloride intracellular channel 6
2.2	24795	Rn.202939	*Serpina3n*	Serpin peptidase inhibitor, clade A (alpha-1 antiproteinase, antitrypsin), member 3
2.2	60665	Rn.44449	*Cxcl6*	Chemokine (C-X-C motif) ligand 6 (granulocyte chemotactic protein 2)
2.1	289546	Rn.136778	*Tmprss11 g*	Transmembrane protease, serine 11 g
2.1	25424	Rn.92738	*Ctse*	Cathepsin E
2.0	407762	Rn.154794	*Krt85*	Keratin 85
2.0	295176	Rn.178258	*Cd79a*	Cd79a molecule, immunoglobulin-associated alpha
1.9	311793	Rn.162651	*Noxa1*	NADPH oxidase activator 1
1.9	81779	Rn.32256	*Sctr*	Secretin receptor
1.9	311803	Rn.154622	*Lrrc26*	Leucine-rich repeat containing 26
1.9	289949	Rn.38497	*Fam3d*	Family with sequence similarity 3, member D
1.8	29527	Rn.44369	*Ptgs2*	Prostaglandin-endoperoxide synthase 2 (prostaglandin G/H synthase and cyclooxygenase)
1.8	65162	Rn.88380	*Dio2*	Deiodinase, iodothyronine, type II
1.8	25098	Rn.10470	*Foxa1*	Forkhead box A1
1.8	619558	Rn.162560	*Fam134b*	Family with sequence similarity 134, member B
1.8	688684	Rn.198280	*LOC688684*	Similar to 60S ribosomal protein L32
1.8	368066	Rn.19133	*Inmt*	Indolethylamine N-methyltransferase

All *P* < 0.05.

Ingenuity Pathway Analysis (IPA) was next used to identify and place all the DEG into different function and disease categories. This confirmed that the main canonical pathways and bio-functions affected related to immune and inflammatory responses and cellular reorganization (Table4). Sub-pathways with the greatest number of molecules represented included glucocorticoid receptor signaling, LXR/RXR activation, Graft-versus-host disease signaling, allograft rejection signaling and Cytotoxic T-Lymphocyte-mediated Apoptosis of Target Cells and Agranulocyte Adhesion and Diapedesis (Table5). All these sub-pathways are consistent with processes associated with immune and inflammatory responses.

**Table 4:** Main functions identified using IPA (all with *P* value <0.001) with differential expression in laboring uterine horn between YOUNG and OLDER rats

Top canonical pathways	Ratio
Graft-versus-Host disease signaling	5/51 (0.098)
LXR/RXR activation	7/139 (0.05)
Granulocyte adhesion and diapedesis	8/181 (0.044)
FXR/RXR activation	6/110 (0.055)
Agranulocyte adhesion and diapedesis	8/191 (0.042)

Top biological functions	Molecules
*Diseases and disorders*	
Cancer	95
Inflammatory response	53
Neurological disease	46
Cardiovascular disease	42
Organismal injury and abnormalities	40
*Molecular and cellular functions*	
Cellular growth and proliferation	73
Cell to cell signaling/interaction	54
Cellular movement	53
Molecular transport	52
Protein synthesis	39
*Physiological system development and function*	
Hematological system development/function	60
Organismal development	55
Tissue morphology	47
Cardiovascular system development and function	47
Immune cell trafficking	39

IPA, ingenuity pathway analysis.

**Table 5:** Top 20 canonical subpathways from IPA analysis (all *P* < 0.001) associated with immune or inflammatory response and lipid transport and metabolism differentially expressed in laboring uterine horn with increasing maternal age

Subpathway	Gene symbol
Graft-versus-Host disease signaling	*Prf1, **HLA-C**, **Il1rn**, HLA-B, **HLA-DQB1**, Gzmb*
LXR/RXR activation	*Ttr, Apob, Apom, Apoh, **Il1rn,** Apoc2, **Ptgs2***
Granulocyte adhesion and diapedesis	***Cxcl3,** Cxcl11, Mmp3, **Il1rn,** Cxcl14, Ccl21, mmp12, Cxcl6*
FXR/RXR activation	*Pparg, Apob, **Il1rn**, **Foxa1**, Apoc2, Mttp*
Agranulocyte adhesion and diapedesis	***Cxcl3**, Cxcl11, Mmp3, **Il1rn**, Cxcl14, Ccl21, mmp12, Cxcl6*
Autoimmune thyroid disease signaling	*Prf1, **HLA-B, HLA-DQB1,** Gzmb*
Allograft rejection signaling	*H2-T24, Prf1, **HLA-B**, **HLA-DQB1**, Gzmb*
Cytotoxic T-lymphocyte-mediated apoptosis of target cells	*H2-T24, Prf1, **HLA-B**, **HLA-DQB1**, Gzmb*
Glucocorticoid receptor signaling	***Cxcl3**, **Scgb1a1,** Taf7 l, **Il1rn,** Plau, **Ptgs2,** Nppa, Cdkn1c,*
Role of IL-17A in psoriasis	***Cxcl3,** Cxcl6*
Atherosclerosis signaling	*Apob, Apom, Mmp3, **Il1rn**, Apoc2*
Oncostatin M signaling	*Mmp3, Plau, Chi3 l1,*
Inhibition of matrix metalloproteases	*Adam12, Mmp3, mmp12,*
Clathrin-mediated endocytosis signaling	*Apob, Apom,**Ephb2**, Figf, Apoc2, Fgf7*
Altered T-cell and B-cell signaling in rheumatoid arthritis	***Il1rn**, Ccl21,**HLA-DQB1**, **Cd79a***
Bladder cancer signaling	*MMP Mmp33, Figf, mmp12, Fgf7,*
Granzyme B signaling	*Prf1, Gzmb*
Role of IL-17A in arthritis	***Cxcl3, Ptgs2, Cxcl6,***
Type I diabetes mellitus signaling	*Prf1,HLA-B,**HLA-DQB1**, Gzmb*
B cell development	***HLA-DQB1**, **Cd79a***

Genes in bold typeface were increased in laboring uterine horn from 6 month (OLDER) rat dams compared with YOUNG animals. Other genes were decreased.

The genes associated with the top five key networks identified by IPA (score >26) are listed in Table6 and illustrated in Figures S1–S5.

**Table 6:** Top 5 networks of DEG in laboring uterine horn between YOUNG and OLDER rat dams

Network	Score	Focus molecules	Molecules in network
1. Cell-To-Cell Signaling and Interaction, Cellular Movement, Immune Cell Trafficking	40	21	*Akt, ALT, Bnip3, Ccl21, Cdkn1c, Ceacam11 (includes others), chemokine, CP, Cxcl3, Cxcl6, Cxcl11, Cxcl14, Fcer1, Foxo4, GC-GCR dimer, GOT, Granzyme, Gzmc (includes others), Gzmf, HLA-DQB1, IL12 (family), Il17r, Il1rn, Interferon alpha, Mttp, N-cor, Pi3k (family), Pik3ip1, Prf1, Prl4a1, Scgb1a1, Sema6d, Tlr, Tnf (family), Tnfrsf9*
2. Endocrine System Development and Function, Small Molecule Biochemistry, Cardiovascular System Development and Function	28	16	*Adcy, Adrb, Calcineurin protein(s), Cg, Cited1, Col15a1, Col8a1, Creb, Cyclin D, Cyp11a1, Dio2, E2f, Endothelin, ERK, Fam3d, Fsh, Fstl3, Gcgr, Gucy, Gucy1b3, Hsd17b2, Lh, Map2k1/2, Nppa, Nppb, Npr3, Oxt, Pkc(s), PLC, Pp2a, Proinsulin, Rap1, S100b, Slc6a2, TCF*
3. Cellular Movement, Cardiovascular System Development and Function, Organismal Development	26	16	*Aoc1, Chi3 l1, collagen, Collagen Alpha1, Collagen type I, Collagen type II, Collagen type III, Collagen type IV, Collagen(s), elastase, ERK1/2, Fgf7, Fgf, Fibrin, Figf, Integrin, Kallikrein, Laminin, Lum, Mmp3, mmp12, Mmp, NfkB1-RelA, Nrg1, Nuclear factor 1, Olr1, Plau, Prl2c2 (includes others), Ptprz1, Rarres2, Serpina3n, Serpinb5, Serpine2, Stat1/3/5 dimer, trypsin*
4. Cell-To-Cell Signaling and Interaction, Hematological System Development and Function, Inflammatory Response	26	16	*A1cf, Actin, Afp, Anxa5, Apoh, AR, Ca2 + , Calcr, Clic5, Dbi, Dlgap1, Eppin-Wfdc6, Flvcr2, Fxdy3, Gpx3, Hnf4a, Htra3, Icam1, LPA, miR-30c-5p (and other miRNAs w/seed GUAAACA), Mtbp, Noxa1, OVOS/OVOS2, Pemt, Pfdn6, Ptp4a1, Rap2c, S100, Sec61b, Stambp, Tdo2, Tmem140, Tp53, Unc5b, Wfdc1*
5. Lipid Metabolism, Small Molecule Biochemistry, Carbohydrate Metabolism	26	15	*Adam8, Adam28, Adamts15, Ano7, Bag4, C14orf80, Capn13, Cd300e, Chst4, Far2, Gnrhr, Gpnmb, Gsdmd, Gsta4, Metalloprotease, Mfrp, miR-1976 (and other miRNAs w/seed CUCCUGC), miR-3173-5p (and other miRNAs w/seed GCCCUGC), miR-4640-5p (and other miRNAs w/seed GGGCCAG), Mlana, Ndufa3, Nnmt, Pcyt1a, Pla2 g15, Prss21, Psme2, RGD1562525 (includes others), Slc12a1, Slc4a11, Tnf, TRAPPC1, Trappc5, Trappc3 l, Ubc, Vars2*

*Network 1* related to “Cell-To-Cell Signaling and Interaction, Cellular Movement, Immune Cell Trafficking” and featured a number of genes associated with Akt signaling and the adipokine TNF*α*. Several chemokines (*Cxcl3, Cxcl6, Cxcl14, and Ccl21*) were more highly expressed in the older animals (ranging from a 1.5 to 2-fold increase) whereas *Cxcl11* was significantly downregulated twofold. *Il1rn*, a naturally occurring inhibitor of both IL1*α* and IL1*β*, was significantly upregulated 1.5-fold in the OLDER animals. Further DEG within the network that relate to the immune system included Major histocompatability complex class II DQB1 (*HLA-DQB1*) (upregulated in older dams) and granzyme C, (*Gzmc*), granzyme F (*Gzmf*), perforin 1 (*Prf1*), Tumor necrosis factor receptor superfamily 9 (*Tnfrsf9*), semaphorin 6D (*Sema6d*), and prolactin family 4, subfamily a, member 1 (*Prl4a1*), which were all downregulated. Finally a small group of genes related to negative cell cycle signaling were significantly downregulated with increasing maternal age. These included the transcription factor Forkhead Box O4 (*Foxo4*), Phosphoinositol 3 kinase interacting protein 1 (*Pik3ip1*), BCL2/adenovirus E1B 19 kDa interacting protein 3 (*Bnip3*), and cyclin-dependent kinase inhibitor 1C (*Cdkn1c*).

*Network 2* was entitled “Endocrine System Development and Function, Small Molecule Biochemistry, Cardiovascular System Development and Function”. It consisted of genes that play a key role in steroid hormone production and others regulated by steroids which are involved in tissue remodeling and smooth muscle contractility. Cytochrome P450, family 11, subfamily A, polypeptide 1 (*Cyp11a1*) and hydroxysteroid-17*β*-dehydrogenase 2 (*Hsd17b2*) were both downregulated in the OLDER dams. *Cyp11a1* catalyzes the conversion of cholesterol to pregnenolone and is the first and rate-limiting step in the synthesis of steroid hormones (Luu-The 2013). *Hsd17b2* oxidizes estradiol to biologically less active estrone, testosterone to androstenedione, and 20 alpha-dihydroprogesterone to progesterone (Andersson and Moghrabi 1997). Possible evidence of a decreased sensitivity to estradiol in the older animals was provided by the twofold decreased expression of Cbp/P300-Interacting Transactivator, With Glu/Asp-Rich Carboxy-Terminal Domain 1 (*Cited1*). This gene functions as a selective coactivator for estrogen dependent transcription (Yahata et al. 2001). Other DEG in Network 2 that are in part regulated by steroids and which were also significantly downregulated in OLDER dams included follistatin-like 3 (*Fstl3*), S100 calcium binding protein B (*S100b*) and guanylate cyclase 1, soluble beta 3 (*Gucy1b3*) and a few genes involved in smooth muscle contraction, namely natriuretic peptide receptor C (*Npr3*) and its natriuretic peptides A and B (*Nppa, Nppb*), oxytocin (Oxt), and solute carrier family 6 (neurotransmitter transporter), member 2 (*Slc6a2*).

*Network 3* concerned **“**Cellular Movement, Cardiovascular System Development and Function, Organismal Development”. This network contained many genes involved in the breakdown and remodeling of extracellular matrix. These included the matrix metalloproteinases *Mmp3* and *Mmp12* and *Plau*, a serine protease which converts plasminogen to plasmin, which were all downregulated in OLDER dams. Three serine protease inhibitors were also differentially expressed: *Serpina3n* and *Serpinb5* were reduced, whereas *Serpine2* was increased. Other genes which were downregulated in OLDER dams included Lumican (*Lum*) which regulates collagen fibrin organization and two growth factors (FGF7 and FIGF).

*Network 4* was entitled “Cell-To-Cell Signaling and Interaction, Hematological System Development and Function, Inflammatory Response”. This included two of the most highly downregulated genes with increasing maternal age: *Afp* (down 10-fold) and *Apoh* (down sevenfold). *Afp* is a glycoprotein involved in binding and transporting a multitude of ligands such as bilirubin, fatty acids, retinoids, and steroids including estradiol (Payne and Katzenellenbogen 1979; Milligan et al. 1998; Arsenov et al. 2001). *Apoh* is implicated in a variety of physiological pathways including lipoprotein metabolism and coagulation (Schousboe and Rasmussen 1995; McNally et al. 1996; Agostinis et al. 2011). The network also included genes that play key roles in immune and inflammatory responses which were differentially downregulated with increased maternal age. These included *Rap2c*, Member Of RAS Oncogene Family (*Rap2c*), Unc-5 homolog B (*Unc5b*), and Netrin 1 (a ligand for *Unc5b*). Other downregulated genes are involved in extracellular matrix remodeling including HtrA serine peptidase 3 (*Htra3*) and the protease inhibitors WAP four-disulfide core domain 1, & 6 (*Wfdc1 and Eppin-Wfdc6*). Two genes important in reactive oxygen species (ROS) production and detoxification were differentially expressed: NADPH oxidase activator 1 (*Noxa1*) was upregulated twofold whereas glutathione peroxidase 3 (*Gpx3*) was downregulated 1.5-fold with increasing maternal age. FXYD domain containing ion transport regulator 3 (*Fxdy3*) was upregulated threefold and feline leukemia virus subgroup C cellular receptor family, member 2 (*Flvcr2*) was downregulated 1.5-fold in older dams. These genes are both important in ion channel signaling and ion transport (Morrison et al. 1995; Brasier et al. 2004). Finally, large (Drosophila) homolog-associated protein 1 (*Dlgap1*), which encodes a protein that is a part of the scaffold in neuronal cells, was significantly upregulated 1.7-fold in the laboring uterine horn with age.

*Network 5***,** “Lipid Metabolism, Small Molecule Biochemistry, Carbohydrate Metabolism,” housed genes that again relate to immune cell signaling and tissue remodeling. These included a significant upregulation in the mRNA expression of carbohydrate (N-acetylglucosamine 6-O) sulfotransferase 4 (*Chst4*), Cd300e molecule (*Cd300e*), a gene similar to paired-immunoglobulin like type 2 receptor beta (*RGD1562525*), ADAM metallopeptidase domain 28 (*Adam28*), calpain 13 (*Capn13*) and downregulation in glycoprotein (transmembrane) nmb (*Gpnmb*), ADAM metallopeptidase with thrombospondin type 1 motif, 15 (*Adamts15*), and phospholipase A2, group XV (*Pla2 g15*). Many of these genes are known to play a role in immune cell signaling. Lipid metabolism is also an important component of pregnancy and the timing of labor. Fatty acids of the n-3 and n-6 series can modify gestational length (Allen and Harris 2001; Wathes et al. 2007) through altered prostaglandin production (Elmes et al. 2004) and oxytocin signaling (Kim et al. 2012). Glutathione-s-transferase alpha 4 (*Gsta4*) is a DEG within this network closely linked to metabolism of n-6 polyunsaturated fatty acids. *Gsta4* associates with membranes and mitochondria (Gardner and Gallagher 2001) to detoxify and remove the lipid peroxide end product 4-hydroxynonenal (4HNE) from cells (Chapple et al. 2013). The network also included two genes associated with roles in ion channels and ion transporters: Solute Carrier Family 12 (sodium/potassium/chloride transporter) member 1 (*Slc12a1*) (decreased threefold with increasing maternal age) and solute carrier family 4, sodium borate transporter member 11 (*Slc4a11*) (upregulated 1.6-fold).

### qPCR analysis

To validate the array data, uterine samples were analyzed by qPCR for 18 DEG and one reference gene, cyclophilin. The selected list included the top nine genes that were identified as being either upregulated or downregulated in the array analysis. Expression of the reference gene was not altered by maternal age (*P* = 0.95). All the 18 genes tested by qPCR showed similar changes in direction to those found by the array analysis (Table7). In half of these the fold change was also significant (*P* < 0.05) whereas in the remainder the *P* values were all <0.2. These findings provide evidence that the microarray and qPCR platforms are highly correlated. However, it is sometimes found that qPCR does not pick up all the significant differences observed by microarray because the two platforms differ in terms of data generation and statistical analysis. There are differences in how values are generated from the two platforms, normalization, for example, is carried out during microarray data analysis. The statistical analyses are based on the mean fold changes, sample size, and individual variation. These factors may lead to differences in statistical *P* values and resulting significance, especially when sample sizes are small.

**Table 7:** Validation of Affymetrix array data with qPCR. Relative transcripts levels in qPCR (mean ± SEM) were measured in the linear range of fluorescence versus cycle curve

Gene	8 weeks	24 weeks	qPCR	Microarray	Fold change	*P*
	*n *= 4	*n *= 5	Fold change	*P*		
*Afp*	2.25 ± 0.99	0.36 ± 0.33	−6.3	0.086	−10	**0.01**
*Apob*	2.03 ± 0.60	0.31 ± 0.29	−6.5	**0.028**	−8.8	**0.02**
*Apoh*	1.77 ± 0.48	0.37 ± 0.36	−4.8	**0.047**	−6.9	**0.02**
*Ceacam11*	1.77 ± 0.80	0.40 ± 0.24	−4.4	0.113	−4.8	**0.01**
*Apom*	2.08 ± 0.95	0.27 ± 0.26	−7.6	0.080	−4.2	**0.02**
*Ttr*	2.23 ± 1.25	0.18 ± 0.16	−12.7	**0.008**^**#**^	−4.3	**0.03**
*Nrk*	1.54 ± 0.32	0.36 ± 0.25	−4.3	**0.020**	−3.9	**0.01**
*Pramef12*	1.63 ± 0.09	0.40 ± 0.17	−4.0	**0.001**	−3.7	**0.002**
*Slc12a1*	1.79 ± 0.41	0.34 ± 0.25	−5.3	**0.016**	−3.3	**0.02**
*Scgb1a1*	0.21 ± 0.03	1.72 ± 0.42	8.4	**0.001**^**#**^	5.1	**0.0001**
*Fxdy3*	0.40 ± 0.11	1.42 ± 0.59	3.6	0.172	3	**0.004**
*Gabrp*	0.53 ± 0.23	1.31 ± 0.26	2.5	0.066	2.6	**0.001**
*Clic6*	0.26 ± 0.15	1.59 ± 0.66	6.1	0.123	2.2	**0.02**
*Serpina3n*	0.07 ± 0.01	0.16 ± 0.04	2.3	0.121	2.2	**0.0002**
*Cxcl6*	0.71 ± 0.28	1.37 ± 0.21	1.9	0.092	2.2	**0.01**
*Krt85*	0.51 ± 0.09	1.50 ± 0.42	3.0	**0.045**^**#**^	2	**0.01**
*Tmprss11g*	0.54 ± 0.10	1.38 ± 0.51	2.5	0.196	2.1	**0.02**
*Cd79a*	0.39 ± 0.24	1.57 ± 0.23	4.0	**0.009**	2	**0.0002**
*Housekeeping gene*						
Cyclophilin	0.13 ± 0.01	0.14 ± 0.03		0.949		

Comparison of qPCR was by *t*-test, those indicated by # were log transformed to normalize variances.

## Discussion

A number of recent clinical trials have provided evidence that advancing maternal age significantly increases the incidence of prolonged labor and risk of emergency cesarean section (Greenberg et al. 2007; Smith et al. 2008) but the causal mechanism has not yet been fully resolved. The aim of this study was to use a rat model to investigate the effects of maternal age on myometrial contractile function during labor to identify possible mechanisms that may cause poor myometrial activity and dysfunctional labor (Bell et al. 2001; Luke and Brown 2007). The key finding was that both spontaneous and stimulated contractile activity of laboring myometrium was significantly altered by maternal age. The spontaneous contractile activity of laboring myometrium from YOUNG rats was threefold greater than that exhibited in OLDER rats. Increasing myometrial activity through treatment with increasing doses of PGF_2*α*_, carbachol, and phenylephrine, which act via FP acetylcholine and *α*1 adrenergic receptors, respectively, revealed that the myometrial response was also age dependent. Interestingly, myometrial strips from laboring YOUNG rats were not responsive to increasing doses of any of the uterotonic receptor agonists, suggesting that the laboring myometrium was already contracting maximally. In contrast, the laboring myometrium from OLDER rats was more sensitive to all myometrial stimulants and responded with greater myometrial contractile activity with increasing concentration bringing the contractile activity closer to that seen in YOUNG animals. Although the myometrial stimulants used in this study act via different receptors, they all use G-protein-coupled receptors and the cAMP signaling pathway. From these findings it could be speculated that differences in myometrial cAMP signaling may be important (Yuan and López Bernal 2007). It should be noted that OLDER rats in this study were mature rather than aged and myometrial activity may become further altered later in life.

These data support the hypothesis proposed by Greenberg et al. (2007) that the aging myometrium is not contracting effectively and requires treatment with uterotonic agents such as oxytocin or PGF_2*α*_ to markedly improve contractile function during labor. Evidence to support decreased contractile function of the aged myometrium is the indication of a greater need for oxytocin augmentation (Main et al. 2000) and that contractility of myometrial strips from different women aged up to 46 years old decreased significantly with maternal age (Smith et al. 2008). A more recent human study by Arrowsmith et al. (2012) also provided experimental data showing that age decreases uterine contractility but only reaching significance in the nonpregnant state. A key difference between the human studies (Smith et al. 2008; Arrowsmith et al. 2012) and the current investigation is that we did not find multiphasic contractions with increasing maternal age as the myometrial contractile traces showed very regular simple phasic contractions at both ages. This difference may potentially be attributed to the myometrium in the human studies being obtained from nonlaboring patients. In regards to the above reservation there is a limitation of this study in that control experiments were not run on parallel strips to determine the effect of time on myometrial contractile function. While there was no evidence to show a significant effect of time on myometrial contractile activity during the equilibrium and baseline recordings equating to at least 60 min, there is a slight possibility that longer periods of time in the organ bath may produce small decreases in amplitude and frequency of contractions, however, all strips were treated equally and received the same uterotonic agents.

With uterine contractile activity during labor being regulated through increased expression of contractile associated proteins it was important to determine whether the age associated decrease in myometrial contractile activity within this study was associated with downregulation of the key contractile associated proteins PTGS2, CAV-1, and GJA1. PTGS2 rises significantly with the onset of labor (Zuo et al. 1994; Dong et al. 1996; Lye 1996) and increases synthesis of the prostaglandins PGE_2_ and PGF_2*α*_, which are central to the mechanism of labor. PGE_2_ causes cervical ripening and uterine relaxation (Lopez Bernal et al. 1993) whereas PGF_2*α*_ stimulates activation of the myometrial contractile machinery that expels the fetus (Challis et al. 1997). Increased CAV-1 and decreased GJA1 protein expression in the myometrium adversely affects myometrial contractile activity (Riley et al. 2003; Doring et al. 2006; Noble et al. 2006). Western blot analysis of all three contractile associated proteins revealed that their uterine expression was unaffected by maternal age. When paralleled with no significant difference in plasma concentrations of PGE_2_, PGF_2*α*_, and progesterone this finding is not unexpected, although *Ptgs2* gene expression was 1.8-fold higher in the OLDER rats. Functional withdrawal of progesterone in the myometrium at the end of pregnancy induces contractile associated protein expression and activates myometrial contractile activity (Lye et al. 1998). This suggests that advancing maternal age is not associated with a change in the expression of contractile proteins per se, but that alternative pathways regulate the differences in contractility.

It has been well documented that cholesterol concentrations increase with age due to altered metabolism (Paik et al. 2013). Cholesterol has recently been shown to depress or inhibit myometrial contractility (Noble et al. 2006; Zhang et al. 2007). In this study, plasma and uterine tissue concentrations of cholesterol and triglycerides were not significantly different between YOUNG and OLDER rats although there was evidence from the array analysis that some pathways involved with cholesterol metabolism were altered.

Having ruled out some of the possible mechanisms which might alter myometrial contractility with age, we next measured global gene expression patterns using microarrays to provide insight into which other pathways might be important. The main canonical pathways and biological functions that were affected by maternal age related to immune and inflammatory responses, lipid transport and metabolism, steroid metabolism, tissue remodeling, and smooth muscle contraction. It should be noted that the tissue analyzed was whole uterine horn, so the DEG might have been located in myometrium or endometrium and both would have included immune cell populations.

Inflammation is a key regulator of the timing of parturition (Norman et al. 2007). Leukocytes (largely neutrophils, macrophages, and T cells including natural killer (NK) cells) invade the myometrium, cervix, and fetal membranes at the onset of labor (Thomson et al. 1999; Osman et al. 2003; Yellon et al. 2003; Gomez-Lopez et al. 2010). This invasion is stimulated by increased tissue expression of chemokines and cell adhesion molecules (Winkler et al. 1998; Ledingham et al. 2001). The invading leukocytes cause a rise in proinflammatory cytokines (Young et al. 2002) that stimulate myometrial contractility and tissue remodeling (Sennstrom et al. 2000). This attracts further leukocytes in a positive feedback mechanism that augments the process of parturition (Elliot et al. 2000).

TNF*α* is a proinflammatory adipokine produced chiefly by macrophages and monocytes (Matthews 1981; Vassalli 1992). A number of genes associated with TNF signaling were differentially expressed in Network 1 with notable increases in uterine expression of the chemokines *Cxcl3, Cxcl6, Cxcl14, and Ccl21* in the OLDER dams. These in turn drive the inward migration of monocytes and macrophages to the site of inflammation and their subsequent activation (Chevillard et al. 2007). Il1*β* is another important cytokine whose increased expression in the cervix and myometrium before or during parturition is thought to contribute to leukocyte infiltration s during labor (Thomson et al. 1999; Haddad et al. 2006; Mittal et al. 2010). IL1RN is a naturally occurring inhibitor of both IL1*α* and IL1*β* (Arend et al. 1998) whose expression declines rapidly before parturition in women (Heng et al. 2014), thus potentially augmenting the actions of IL-1 in the myometrium and cervix (Romero et al. 1992; Brown et al. 1998). In Network 1 *Il1rn* showed a significant upregulation in the older dams, suggesting that this augmentation of the myometrium to interleukins could be suppressed.

Other components of Network 1 suggested alterations in T-cell responses with increasing age, with a significant reduction in expression of the following genes. *HLA-DQB1* binds peptides derived from antigens on their cell surface for recognition by CD4 T cells, driving activation of a T-cell response (Braciale et al. 1987). Once activated, cytoxic T cells and NK cells release granules containing granzymes (*Gzmc* and *Gzmf*) and *Prf1*. *Prf1* helps to create pores within the cell membrane of target cells through which the granzymes, which are serine proteases, can enter and induce apoptosis (Grossman et al. 2003; Bots and Medema 2006). *Tnfrsf9* is a receptor that contributes to the clonal expansion, survival, and development of T cells and is expressed in immune cells including placental macrophages (Phillips et al. 2001). *Tnfrsf9* expression increases dramatically in the murine uterus during implantation but decreases between days 13 and 19 of gestation (Zhao et al. 2007; Eckstrum and Bany 2011). This downregulation may help maintain a successful pregnancy (Sykes et al. 2012) as its blockade increases allograft survival in cardiac transplants. *Sema6d* also plays a role in T-cell activation as targeted disruption of Sema6d ligand interactions inhibit T-cell proliferation (O'Connor et al. 2008).

Interestingly *Prl4a1* encodes the hormone prolactin which is a growth regulator of many tissues including cells of the immune system. Prolactin is expressed in human T lymphocytes and is regulated by cytokines (Gerlo et al. 2005). It is possible that the fourfold downregulation in *Prl4a1* expression indicates a lower density of lymphocytes in the uterine horn during labor with increasing maternal age. On the other hand, *Chst4* (Network 5) is an enzyme which plays a central role in lymphocyte trafficking and Chst4 deficient mice have a 60% decrease in lymphocytes number (Hemmerich et al. 2001; van Zante et al. 2003). Increased *Chst4* expression in older animals would therefore be expected to increase lymphocyte numbers. Together these changes in gene expression suggest that the uterus of the OLDER rats showed decreased inflammation at labor onset in comparison with the YOUNG animals.

Networks 4 and 5 also contained many DEG that play key roles in immune and inflammatory responses which were altered with maternal age. *Rap2c* is a small GTPase found predominantly in leukocytes that acts as a molecular switch to regulate cell proliferation and apoptosis (Paganini et al. 2006). *Unc5b* is a gene that encodes a netrin receptor that is expressed on granulocytes, monocytes, and lymphocytes (Ly et al. 2005). Binding of Netrin 1 causes a significant decrease in cytokine and chemokine production (Tadagavadi et al. 2010) and also blocks migration of macrophages (Ramkhelawon et al. 2014). *Cd300e* is a glycoprotein expressed in monocytes which acts as an activating receptor to induce expression of the pro-flammatory cytokines and chemokines IL-8, CXCL8, and TNF*α* (Brckalo et al. 2010). The paired-immunoglobulin like type 2 family of receptors consists of both activating and inhibiting receptors. The beta isoform is primarily expressed by NK cells (Shiratori et al. 2004) and a deficiency in mice with chronic inflammation promoted IL10 and IL27 production in effector T cells (Tato et al. 2012). Therefore, an increase in the beta receptor (*RGD1562525*) in older animals may suggest decreased production of interleukins. *ADAM28* encodes a member of the ADAM (a disintegrin and metalloprotease domain) family of proteins which are highly expressed in T and B lymphocytes (McGinn et al. 2011). *ADAM28* influences lymphocyte adhesion (Roberts et al. 1999) and migration (McGinn et al. 2011). *Pla2 g15* deficiency results in impaired NK cell development and this gene was downregulated 1.5-fold in OLDER uterine tissue also suggesting a dampening of an immune response with age.

Several of the genes with the greatest fold changes in expression were involved in lipid metabolism, transport, and cholesterol homoeostasis. This contrasts with our finding that maternal age had no significant effect on triglyceride and cholesterol content within the uterine horn. Uterine tissue from OLDER animals exhibited a large fold decrease in expression of *Apob, Apoc2, Apoh,* and *Apom*. *Apoh* is implicated in a variety of physiological pathways including binding to endothelium (Agostinis et al. 2011) and exhibiting anticoagulation properties (Schousboe and Rasmussen 1995; McNally et al. 1996). The cholesterol concentration increases greatly during pregnancy (Toescu et al. 2004) and is the precursor for synthesis of the steroid hormones progesterone and estradiol (Elovitz and Wang 2004) although cholesterol itself can decrease myometrial contractile activity (Smith et al. 2005). Glucorticoid receptor signaling and LXR/RXR activation all occur through the nuclear receptor superfamily and have regulatory roles in inflammatory processes. Glucocorticoid receptor agonists are used clinically to inhibit inflammatory diseases (Coutinho and Chapman 2011). LXRs play a key role in maintaining cholesterol homeostasis in macrophages and regulate their inflammatory pathways. Mice lacking LXRs show an exaggerated response to both lipopolysaccharide and synthetic LXR agonists which inhibit macrophage responses to bacterial pathogens and antagonize the induction of a number of pro-inflammatory genes (Castrillo et al. 2003; Joseph et al. 2003b).

Network 2 consisted of genes that play a role in steroid hormone production and genes regulated by steroids which are involved in tissue remodeling and smooth muscle contractility. *Cyp11a1 and Hsd17b2* were both downregulated in the uterus of the OLDER rats during labor. *Cyp11a1* catalyzes the conversion of cholesterol to pregnenolone and is the first and rate-limiting step in the synthesis of steroid hormones (Luu-The 2013). *Hsd17b2* oxidizes estradiol to biologically less active estrone, testosterone to androstenedione, and 20 alpha-dihydroprogesterone to progesterone (Andersson and Moghrabi 1997). Downregulation of *Cyp11a1* in OLDER uterine tissue suggests a decreased capacity to produce steroid hormones from cholesterol, whereas the decreased expression of *Hsd17b2* may be an adaptation to increase production of the more active steroids, particularly estradiol. There was also a twofold decreased expression of *Cited1* in the older animals. This gene enhances tissue sensitivity to estrogen (Yahata et al. 2001) so our results suggest there may be decreased sensitivity to estradiol in the OLDER rats. Network 4 contained *Afp*, the gene that was most downregulated by 10-fold with increasing maternal age. *Afp* is a glycoprotein involved in binding and transporting a multitude of ligands such as bilirubin, fatty acids, retinoids, and steroids (Milligan et al. 1998; Arsenov et al. 2001). In rats and mice *Afp* binds estradiol with high affinity (Payne and Katzenellenbogen 1979; Garreau et al. 1991) and can decrease production of PGE_2_ in the human placenta (Aussel 1984).

In parallel to this a number of genes encoding proteins which are potentially involved in tissue remodeling of the uterus and which exhibit some control by estradiol were significantly downregulated with increasing maternal age (*Fstl3, S100b, Gucy1b3*) although *Dlgap1* expression was higher. Uterine mRNA expression of *Fstl3* is low during early pregnancy but continuously increases during the second half of gestation (Arai et al. 2003) Its expression is induced by estradiol in combination with progesterone (Wang et al. (2003) *S100b* is significantly increased following amniotic infection and inflammation associated with premature labor (Friel et al. 2007). *Dlgap1* encodes a protein that is a part of the scaffold in neuronal cells. It is well established that pregnancy is accompanied by axonal degeneration in rats and guinea pigs (Klukovits et al. 2002; Richeri et al. 2005) and the mechanism is through increasing serum levels of estradiol (Zoubina and Smith 2000).

A large number of genes involved in tissue remodeling were differentially expressed with maternal age. These mainly appeared in Networks 3 and 4. Fibroblasts orchestrate ECM remodeling of the uterus and cervix during pregnancy (Malmström et al. 2007). In the OLDER rats there was a three- and twofold downregulation of *Mmp3* and *Mmp12,* respectively, which both degrade the extracellular matrix, a process key to parturition (Morgan et al. 1998). Expression of *Gpnmb* was also lower; this protein is produced in differentiated immune cells and increase *Mmp3* expression (Ogawa et al. 2005). In addition there was differential expression of serpins, the key inhibitors of proteases (Ebisch et al. 2008). The uterine horn of OLDER animals had decreased gene expression of *Serpine2* but increased expression of *Serpina3n* and *Serpinb5*. The serine protease *Plau* which converts plasminogen to plasmin was twofold lower in OLDER rat uterus and *Capn13*, calcium activated protease, was 1.7-fold higher in the OLDER rats. *Capn13* activity in the rat uterus is lowest in the nonpregnant state but then increases throughout pregnancy peaking at term and 1 day postpartum (Elce et al. 1984). Another protease *Htra3* was also downregulated. This is present in the endometrium and placenta during early pregnancy (Nie et al. 2003) and has an inhibitory effect on trophoblast invasion *in vitro* (Singh et al. 2011). Additional downregulated genes associated with tissue remodeling include *Wfdc1 and Eppin-Wfdc6* which function as protease inhibitors. They are highly expressed in uterine smooth muscle (Larsen et al. 1998; Hung 2005) and uterine *Wfdc1* levels fall in pro-estrous and rise during diestrous, suggesting steroidal regulation (Hung 2005). Overall these findings suggest that remodeling of the uterine tissue may be delayed in the OLDER uterine horn during labor. Although many of these changes may relate to endometrium rather than myometrium, they may nevertheless contribute to the adverse effects on myometrial contractility which we observed in the same tissues.

A small number of genes that were downregulated in uterine tissue of the OLDER laboring animals are involved in smooth muscle contraction including natriuretic peptides A and B (*Nppa, Nppb*) and the natriuretic peptide receptor C (*Npr3*). Binding to Npr3 induces relaxation of smooth muscle via conversion of GTP to cGMP. Importantly, the cGMP content in myometrium obtained during premature delivery is significantly higher than that of nonpregnant myometrium and it decreases at term especially during labor (Telfer et al. 2001). The decrease in expression of *Npr3* and its natriuretic peptides along with reduced availability of cGMP may regulate the switch from quiescence to contractile activity at term. Both *Oxt* and *Slc6a2* were also significantly downregulated with age. Oxytocin increases both the frequency and force of smooth muscle contractions during parturition and pharmacological inhibition of oxytocin action delays delivery in rats and guinea pigs (Chan and Chen 1992; Schellenberg 1995). *Slc6a2* is a multipass protein that terminates the action of noradrenaline. Inhibition of this transporter leads to myometrial vasoconstriction and uterine contractility (Pennefather et al. 1993). *Gsta4* was downregulated 1.5-fold. This protein protect mitochondria and cells from the toxic effects of 4HNE an *α*,*β*-unsaturated hydroxyalkenal that is produced by lipid peroxidation (Chapple et al. 2013) and which increases in plasma and tissues with age (Gil et al. 2006). Furthermore, 4HNE also upregulates myometrial mRNA expression of *Ptgs2* (a gene also increased 1.8-fold in this study) and PGE_2_ synthesis (a hormone that promotes uterine relaxation) in a dose-dependent manner (Temma-Asano et al. 2011).

Network 1 also included a small group of genes that were all significantly downregulated with increasing maternal age that relate to negative cell cycle signaling: *Foxo4, Pik3ip1, Bnip3,* and *Cdkn1c*. *Foxo4* acts downstream of the PI3K/AKT signaling pathway, whereas *Pik3ip1* inhibits the PI3K pathway and both genes suppress cell proliferation and differentiation (Zhu et al. 2007). *Bnip3* is an apoptosis inducing protein which is decreased in pregnancies associated with placental dysfunction and hypertensive disorders (Stepan et al. 2005). *Cdkn1c* is also a negative regulator of cell proliferation by causing cell cycle arrest in the G1 phase (Lee et al. 1995).

Network 2 contained two genes which are important in reactive oxygen species (ROS) production and detoxification: *Noxa1* was upregulated whereas *Gpx3* was downregulated with increasing maternal age. Proposed functions of *Noxa1* in the uterus include activation of NFK*β* leading to PGF_2*α*_ production (Sugino et al. 2004) and angiogenesis during the menstrual cycle (Agarwal et al. 2005). *Gpx3* does the opposite to *Noxa1* by functioning to detoxify hydrogen peroxide and lipid peroxides and it acts as a redox buffer against inflammatory stimuli (Brigelius-Flohé 1999).

Some genes involved in ion channel signaling and ion transport were also differentially expressed with increasing maternal age. *Fxdy3* was upregulated and *Flvcr2* was downregulated. *Fxdy3* is expressed in high levels in the uterus (Morrison et al. 1995) and is a protein key to regulating ion pumps and channels (Morrison et al. 1995). *Flvcr2* encodes a transmembrane calcium transporter (Brasier et al. 2004) which also acts as an importer of heme (Duffy et al. 2010). In Network 3 *Slc12a1* decreased, whereas *Slc4a11* was upregulated with increasing maternal age. *Slc12a1* plays a role in kidney function by mediating sodium and chloride resorption (Simon et al. 1996; Ares et al. 2011). *Slc4a11* has been characterized as a sodium-coupled borate cotransporter essential for cell growth and proliferation (Park et al. 2004).

In conclusion, the prevalence of advanced maternal age for first time pregnant mothers is steadily increasing and is associated with prolonged and dysfunctional labor and increased risk of cesarean section. This is the first study to determine that the contractile activity of laboring myometrial tissue in rats is adversely affected by a modest increase in maternal age. OLDER animals showed less spontaneous contractile activity and a greater response to increasing doses of myometrial contractile agents. These responses were absent in YOUNG laboring tissue as this was closer to contracting maximally without artificial stimulation. Our physiological data thus confirm that older women may require uterotonic agents to help stimulate myometrial activity where labor is progressing slowly, and that higher doses of uterotonic agents may be required for labor induction in the aged compared to young myometrium. We have also identified a number of possible pathways that may contribute to this adverse age effect including suppression of immune responses and inflammation, altered steroid metabolism, and altered or incomplete uterine tissue remodeling. Further research into the effects of maternal age on myometrial contractile function is warranted, particularly the involvement of lipid metabolism and steroid production on inflammatory pathways and remodeling of the term and laboring uterus.

## Supporting Information

**Table S1.** Oligonucleotide primer sequence for quantitative real-time PCR assays.

**Table S2.** The 129 genes that were significantly different between the older and younger rats.

**Figure S1.** IPA network 1.

**Figure S2.** IPA network 2.

**Figure S3.** IPA network 3.

**Figure S4.** IPA network 4.

**Figure S5.** IPA network 5.

## References

[b2] Agarwal A, Gupta S, Sharma RK (2005). Role of oxidative stress in female reproduction. Reprod. Biol. Endocrinol.

[b3] Agostinis C, Biffi S, Garrovo C, Durigutto P, Lorenzon A, Bek A (2011). In vivo distribution of *β*2 glycoprotein I under various pathophysiologic conditions. Blood.

[b4] Allen KG, Harris MA (2001). The role of n-3 fatty acids in gestation and parturition. Exp. Biol. Med. (Maywood).

[b5] Andersson S, Moghrabi N (1997). Physiology and molecular genetics of 17 beta-hydroxysteroid dehydrogenases. Steroids.

[b6] Arai KY, Tsuchida K, Uehara K, Taya K, Sugino H (2003). Characterization of rat follistatin-related gene: effects of estrous cycle stage and pregnancy on its messenger RNA expression in rat reproductive tissues. Biol. Reprod.

[b7] Arend WP, Malyak M, Guthridge CJ, Gabay C (1998). Interleukin-1 receptor antagonist: role in biology. Annu. Rev. Immunol.

[b8] Ares GR, Caceres PS, Ortiz PA (2011). Molecular regulation of NKCC2 in the thick ascending limb. Am. J. Physiol. Renal. Physiol.

[b9] Arrowsmith S, Robinson H, Noble K, Wray S (2012). What do we know about what happens to myometrial function as women age?. J. Muscle Res. Cell Motil.

[b10] Arsenov DV, Golubeva MB, Kisel’ MA, Konoplya NA, Lyubin GS, Kuz'mitskii BB (2001). Modification of humoral immune response in C57Bl/6 mice with a complex of alpha-fetoprotein and retinoid acid derivatives. Bull. Exp. Biol. Med.

[b11] Aussel C (1984). Prostaglandin synthesis in human placenta and fetal membranes: partial inhibition by rat and human alpha-fetoproteins. Res. Commun. Chem. Pathol. Pharmacol.

[b12] Babiychuk EB, Smith RD, Burdyga T, Babiychuk VS, Wray S, Draeger A (2004). Membrane cholesterol regulates smooth muscle phasic contraction. J. Membr. Biol.

[b13] Bakircioglu ME, Sievert KD, Nunes L, Lau A, Lin CS, Lue TF (2001). Decreased trabecular smooth muscle and caveolin-1 expression in the penile tissue of aged rats. J. Urol.

[b14] Bell JS, Campbell DM, Graham WJ, Penney GC, Ryan M, Hall MH (2001). Can obstetric complications explain the high levels of obstetric interventions and maternity service use among older women? A retrospective analysis of routinely collected data. BJOG.

[b15] Bots M, Medema JP (2006). Granzymes at a glance. J. Cell. Sc.

[b16] Braciale TJ, Morrison LA, Sweetser MT, Sambrook J, Gething MJ, Braciale VL (1987). Antigen presentation pathways to class II MHC-restricted T lymphocytes. Immunol. Rev.

[b17] Brasier G, Tikellis C, Xuereb L, Craigie J, Casley D, Kovacs CS (2004). Novel hexad repeats conserved in a putative transporter with restricted expression in cell types associated with growth, calcium exchange and homeostasis. Exp. Cell Res.

[b18] Brckalo T, Calzetti F, Pérez-Cabezas B, Borràs FE, Cassatella MA, López-Botet M (2010). Functional analysis of the CD300e receptor in human monocytes and myeloid dendritic cells. Eur. J. Immunol.

[b19] Bréart G, Barros H, Wagener Y, Prati S (2003). Characteristics of the childbearing population in Europe. Eur. J. Obstet. Gynecol. Reprod. Biol.

[b20] Brigelius-Flohé R (1999). Tissue-specific functions of individual glutathione peroxidases. Free Radic. Biol. Med.

[b21] Brown NL, Alvi SA, Elder MG, Bennett PR, Sullivan MH (1998). Regulation of prostaglandin production in intact fetal membranes by interleukin-1 and its receptor antagonist. J. Endocrinol.

[b22] Castrillo A, Joseph SB, Narathe C, Mangelsdorf DJ, Tonontoz P (2003). Liver X receptor dependent repression of matrix metalloproteinase-9 expression in macrophages. J. Biol. Chem.

[b23] Challis JR, Lye SJ, Gibb W (1997). Prostaglandins and parturition. Ann. N. Y. Acad. Sci.

[b24] Chan WY, Chen DL (1992). Myometrial oxytocin receptors and prostaglandin in the parturition process in the rat. Biol. Reprod.

[b25] Chapple SJ, Cheng X, Mann GE (2013). Effects of 4-hydroxynonenal on vascular endothelial and smooth muscle cell redox signaling and function in health and disease. Redox. Biol.

[b26] Chevillard G, Derjuga A, Devost D, Zingg HH, Blank V (2007). Identification of interleukin-1beta regulated genes in uterine smooth muscle cells. Reproduction.

[b27] Cluff AH, Bystrom B, Klimaviciute A, Dahlqvist C, Cebers G, Malmstrom A (2006). Prolonged labour associated with lower expression of syndecan 3 and connexin 43 in human uterine tissue. Reprod. Biol. Endocrinol.

[b28] Coutinho AE, Chapman KE (2011). The anti-inflammatory and immune suppressive effects of glucocorticoids, recent developments and mechanistic insights. Mol. & Cell Endo.

[b29] Dong YL, Gangula PR, Fang L, Yallampalli C (1996). Differential expression of cyclooxygenase-1 and -2 proteins in rat uterus and cervix during the estrous cycle, pregnancy, labor and in myometrial cells. Prostaglandins.

[b30] Doring B, Shynlova O, Tsui P, Eckardt D, Janssen-Bienhold U, Hofmann F (2006). Ablation of connexion 43 in uterine smooth muscle cells of the mouse causes delayed parturition. J. Cell Sci.

[b31] Drab M, Verkade P, Elger M, Kasper M, Lohn M, Lauterbach B (2001). Loss of caveolae, vascular dysfunction, and pulmonary defects in caveolin-1 gene-disrupted mice. Science.

[b32] Duffy SP, Shing J, Saraon P, Berger LC, Eiden MV, Wilde A (2010). The Fowler syndrome-associated protein FLVCR2 is an importer of heme. Mol. Cell. Biol.

[b33] Ebisch IM, Thomas CM, Wetzels AM, Willemsen WN, Sweep FC, Steegers-Theunissen RP (2008). Review of the role of the plasminogen activator system and vascular endothelial growth factor in subfertility. Fertil. Steril.

[b34] Eckstrum K, Bany BM (2011). Tumor necrosis factor receptor subfamily 9 (Tnfrsf9) gene is expressed in distinct cell populations in mouse uterus and conceptus during implantation period of pregnancy. Cell Tissue Res.

[b35] Elce JS, Baenziger JE, Young DC (1984). Ca2+-activated proteinase in the rat. Quantification by immunoassay in the uterus during pregnancy and involution, and in other tissues. Biochem. J.

[b36] Elliot CL, Slater DM, Dennes W, Poston L, Bennett PR (2000). Interleukin 8 expression in human mymetrium changes in relation to labor onset and with gestational age. Am. J. Rep. Immunol.

[b37] Elmes M, Tew P, Cheng Z, Kirkup SE, Abayasekara DR, Calder PC (2004). The effect of dietary supplementation with linoleic acid to late gestation ewes on the fatty acid composition of maternal and fetal plasma and tissues and the synthetic capacity of the placenta for 2-series prostaglandins. Biochim. Biophys. Acta.

[b38] Elovitz M, Wang Z (2004). Medroxyprogesterone acetate but not progesterone, protects against inflammation induced parturition and intra-uterine fetal demise. Am. J. Obstet. Gynecol.

[b39] Friel LA, Romero R, Edwin S, Nien JK, Gomez R, Chaiworapongsa T (2007). The calcium binding protein, S100B, is increased in the amniotic fluid of women with intra-amniotic infection/inflammation and preterm labor with intact or ruptured membranes. J. Perinat. Med.

[b40] Gardner JL, Gallagher EP (2001). Development of a peptide antibody specific to human glutathione S-transferase alpha 4-4 (hGSTA4-4) reveals preferential localization in human liver mitochondria. Arch. Biochem. Biophys.

[b41] Garreau B, Vallette G, Adlercreutz H, Wähälä K, Mäkelä T, Benassayag C (1991). Phytoestrogens: new ligands for rat and human alpha-fetoprotein. Biochim. Biophys. Acta.

[b42] Gerlo S, Verdood P, Hooghe-Peters EL, Kooijman R (2005). Modulation of prolactin expression in human T lymphocytes by cytokines. J. Neruroimmunol.

[b43] Gil L, Siems W, Mazurek B, Gross J, Schroeder P, Voss P (2006). Age-associated analysis of oxidative stress parameters in human plasma and erythrocytes. Free Radic. Res.

[b44] Gomez-Lopez N, Guilbert LJ, Olson DM (2010). Invasion of the leukocytes into the fetal-maternal interface during pregnancy. J. Leukoc. Biol.

[b45] Greenberg MB, Cheng YW, Sullivan M, Norton ME, Hopkins LM, Caughey AB (2007). Does length of labor vary by maternal age?. Am. J. Obstet. Gynecol.

[b46] Gross G, Imamura T, Vogt SK, Wozniak DF, Nelson DM, Sadovsky Y (2000). Inhibition of cyclooxygenase-2 prevents inflammation-mediated preterm labor in the mouse. Am. J. Physiol. Regul. Integr. Comp. Physiol.

[b47] Grossman WJ, Revell PA, Lu ZH, Johnson H, Bredemeyer AJ, Ley TJ (2003). The orphan granzymes of humans and mice. Curr. Opin. Immunol.

[b48] Guihard P, Blondel B (2001). Trends in risk factors for caesarean sections in France between 1981 and 1995: lessons for reducing the rates in the future. BJOG.

[b49] Haddad R, Tromp G, Kuivaniemi H, Chaiworapongsa T, Kim YM, Mazor M (2006). Human spontaneous labor without histologic chorioamnionitis is characterized by an acute inflammation gene expression signature. Am. J. Obstet. Gynecol.

[b50] Hamilton BE, Martin JA, Ventura SJ (2009). Births; preliminary data for 2007. National Vital Stat. Rep.

[b51] Hemmerich S, Bistrup A, Singer MS, van Zante A, Lee JK, Tsay D (2001). Sulfation of L-selectin ligands by an HEV-restricted sulfotransferase regulates lymphocyte homing to lymph nodes. Immunity.

[b52] Heng YJ, Liong S, Permezel M, Rice GE, Di Quinzio MK, Georgiou HM (2014). The interplay of the interleukin 1 system in pregnancy and labor. Reprod. Sci.

[b53] Hung H (2005). Suppression of ps20 expression in the rat uterus by tamoxifen and estrogens. Endocrinology.

[b54] Joseph KS, Young DC, Dodds L, O'Connell CM, Allen VM, Chandra S (2003a). Changes in maternal characteristics and obstetric practice and recent increases in primary cesarean delivery. Obstet. Gynecol.

[b55] Joseph SB, Castrillo A, laffitte BA, Mangelsdorf DJ, Tontonoz P (2003b). Reciprocal regulation of inflammation and lipid metabolism by liver X receptors. Nat. Med.

[b56] Keirse MJNC, de Koning Gans HJ (1995). Randomized comparison of the effects of endocervical and vaginal prostaglandin E2 gel in women with various degrees of cervical ripeness. Am. J. Obstet. Gynecol.

[b57] Kim PY, Zhong M, Kim YS, Sanborn BM, Allen KG (2012). Long chain polyunsaturated fatty acids alter oxytocin signaling and receptor density in cultured pregnant human myometrial smooth muscle cells. PLoS ONE.

[b58] King J, Flenady V, Cole S, Thornton S (2005). Cyclo-oxygenase (COX) inhibitors for treating preterm labour. Cochrane Database Syst. Rev.

[b59] Klukovits A, Gáspár R, Sántha P, Jancsó G, Falkay G (2002). Functional and histochemical characterization of a uterine adrenergic denervation process in pregnant rats. Biol. Reprod.

[b60] Kozinszky Z, Orvos H, Zoboki T, Katona M, Wayda K, Pál A (2002). Risk factors for cesarean section of primiparous women aged over 35 years. Acta Obstet. Gynecol. Scand.

[b61] Larsen M, Ressler SJ, Lu B, Gerdes MJ, McBride L, Dang TD (1998). Molecular cloning and expression of ps20 growth inhibitor. A novel WAP-type “four-disulfide core” domain protein expressed in smooth muscle. J. Biol. Chem.

[b62] Ledingham MA, Thomson AJ, Jordan F, Young A, Crawford M, Norman JE (2001). Cell adhesion molecule expression in the cervix and myometrium during pregnancy and parturition. Obstet. Gynecol.

[b63] Lee MH, Reynisdóttir I, Massagué J (1995). Cloning of p57KIP2, a cyclin-dependent kinase inhibitor with unique domain structure and tissue distribution. Genes Dev.

[b64] Leung ST, Cheng Z, Sheldrick EL, Derecka K, Derecka K, Flint AP (2001). The effects of lipopolysaccharide and interleukins- 1alpha, -2 and -6 on oxytocin receptor expression and prostaglandin production in bovine endometrium. J. Endocrinol.

[b65] Lopez Bernal A, Watson SP, Phaneuf S, Europe-Finner GN (1993). Biochemistry and physiology of preterm labour and delivery. Baillieres Clin. Obstet. Gynaecol.

[b66] Luke B, Brown MB (2007). Elevated risks of pregnancy complications and adverse outcomes with increasing maternal age. Hum. Reprod.

[b67] Luu-The V (2013). Assessment of steroidogenesis and steroidogenic enzyme functions. J. Steroid Biochem. Mol. Biol.

[b68] Ly NP, Komatsuzaki K, Fraser IP, Tseng AA, Prodhan P, Moore KJ (2005). Netrin-1 inhibits leukocyte migration in vitro and in vivo. Proc. Natl Acad. Sci. U S A.

[b69] Lye SJ (1996). Initiation of parturition. Anim. Rep. Sci.

[b70] Lye SJ, Ou C-W, Teoh T-G, Erb G, Stevens Y, Casper R (1998). The molecular basis of labour and tocolysis. Fet. Mat. Med. Rev.

[b71] Main DM, Main EK, Moore DH (2000). The relationship between maternal age and uterine dysfunction: a continuous effect throughout reproductive life. Am. J. Obstet. Gynecol.

[b72] Malmström E, Sennström M, Holmberg A, Frielingsdorf H, Eklund E, Malmström L (2007). The importance of fibroblasts in remodelling of the human uterine cervix during pregnancy and parturition. Mol. Hum. Reprod.

[b73] Martin JA, Hamilton BE, Sutton PD, Ventura SJ, Menacker F, Kirmeyer S (2006). Births: final data for 2004. Natl. Vital Stat. Rep.

[b74] Matthews N (1981). Tumour-necrosis factor from the rabbit. V. Synthesis in vitro by mononuclear phagocytes from various tissues of normal and BCG-injected rabbits. Br. J. Cancer.

[b75] McGinn OJ, English WR, Roberts S, Ager A, Newham P, Murphy G (2011). Modulation of integrin *α*4*β*1 by ADAM28 promotes lymphocyte adhesion and transendothelial migration. Cell Biol. Int.

[b76] McNally T, Mackie IJ, Isenberg DA, Machin SJ (1996). beta 2 glycoprotein-I inhibits factor XII activation on triglyceride rich lipoproteins: the effect of antibodies from plasma of patients with antiphospholipid syndrome. Thromb. Haemost.

[b77] Milligan SR, Khan O, Nash M (1998). Competitive binding of xenobiotic oestrogens to rat alpha-fetoprotein and to sex steroid binding proteins in human and rainbow trout (Oncorhynchus mykiss) plasma. Gen. Comp. Endocrinol.

[b78] Mittal P, Romero R, Tarca AL, Gonzalez J, Draghici S, Xu Y (2010). Characterization of the myometrial transcriptome and biological pathways of spontaneous human labor at term. J. Perinat. Med.

[b79] Montan S (2007). Increased risk in the elderly parturient. Curr. Opin. Obstet. Gynecol.

[b80] Morgan M, Kniss D, McDonnell S (1998). Expression of metalloproteinases and their inhibitors in human trophoblast continuous cell lines. Exp. Cell Res.

[b81] Morrison BW, Moorman JR, Kowdley GC, Kobayashi YM, Jones LR, Leder P (1995). Mat-8, a novel phospholemman-like protein expressed in human breast tumors, induces a chloride conductance in Xenopus oocytes. J. Biol. Chem.

[b82] Nie GY, Li Y, Minoura H, Batten L, Ooi GT, Findlay JK (2003). A novel serine protease of the mammalian HtrA family is up-regulated in mouse uterus coinciding with placentation. Mol. Hum. Reprod.

[b83] Noble K, Zhang J, Wray S (2006). Lipid rafts, the sarcoplasmic reticulum and uterine calcium signalling: an integrated approach. J. Physiol.

[b84] Norman JE, Bollapragada S, Yuan M, Nelson SM (2007). Inflammatory pathways in the mechanism of parturition. BMC Preg. & Childbirth.

[b85] O'Connor BP, Eun S-Y, Ye Z, Zozulya AL, Lich JD, Moore CB (2008). Semaphorin 6D regulates the late phase of CD4+ T cell primary immune responses. PNAS.

[b86] Ogawa T, Nikawa T, Furochi H, Kosyoji M, Hirasaka K, Suzue N (2005). Osteoactivin upregulates expression of MMP-3 and MMP-9 in fibroblasts infiltrated into denervated skeletal muscle in mice. Am. J. Physiol. Cell Physiol.

[b87] Okamoto T, Schlegel A, Scherer PE, Lisanti MP (1998). Caveolins, a family of scaffolding proteins for organizing “preassembled signaling complexes” at the plasma membrane. J. Biol. Chem.

[b88] Osman I, Young A, Ledingham MA, Thompson AJ, Jordan F, Greer IA (2003). Leukocyte density and pro-inflammatory cytokine expression in human fetal membranes, decidua, cervix and myometrium, before and during labour at term. Mol. Hum. Reprod.

[b89] Paganini S, Guidetti GF, Catricalà S, Trionfini P, Panelli S, Balduini C (2006). Identification and biochemical characterization of Rap2C, a new member of the Rap family of small GTP-binding proteins. Biochimie.

[b90] Paik JK, Chae JS, Kang R, Kwon N, Lee S-H, Lee JH (2013). Effect of age on atherogenicity of LDL and inflammatory markers in healthy women. Nutr. Metab. Cardiovasc. Dis.

[b91] Park M, Li Q, Shcheynikov N, Zeng W, Muallem S (2004). NaBC1 is a ubiquitous electrogenic Na+ -coupled borate transporter essential for cellular boron homeostasis and cell growth and proliferation. Mol. Cell.

[b92] Payne DW, Katzenellenbogen JA (1979). Binding specificity of rat alpha-fetoprotein for a series of estrogen derivatives: studies using equilibrium and nonequilibrium binding techniques. Endocrinology.

[b93] Pennefather JN, Paull JD, Story ME, Ziccone SP (1993). Supersensitivity to the stimulant action of noradrenaline on human myometrium near term. Reprod. Fertil. Dev.

[b94] Phillips TA, Ni J, Hunt JS (2001). Death-inducing tumour necrosis factor (TNF) superfamily ligands and receptors are transcribed in human placentae, cytotrophoblasts, placental macrophages and placental cell lines. Placenta.

[b95] Quaas L, Zahradnik HP, Breckwoldt M (1985). Age-, cycle- and topographic dependency of human myometrial prostaglandin (6-keto-PGF1 alpha, PGF2 alpha)-synthesis in vitro. Prostaglandins.

[b96] Ramkhelawon B, Hennessy EJ, Ménager M, Ray TD, Sheedy FJ, Hutchison S (2014). Netrin-1 promotes adipose tissue macrophage retention and insulin resistance in obesity. Nat. Med.

[b97] Richeri A, Bianchimano P, Mármol NM, Viettro L, Cowen T, Brauer MM (2005). Plasticity in rat uterine sympathetic nerves: the role of TrkA and p75 nerve growth factor receptors. J. Anat.

[b98] Riley M, Baker P, Taggart M (2003). Effects of methyl-*β*-cyclodextrin on spontaneous and oxytocin-induced contractility of isolated human uterine smooth muscle. J. Physiol.

[b99] Roberts CM, Tani PH, Bridges LC, Laszik Z, Bowditch RD (1999). MDC-L, a novel metalloprotease disintegrin cysteine-rich protein family member expressed by human lymphocytes. J. Biol. Chem.

[b100] Romero R, Sepulveda W, Mazor M, Brandt F, Cotton DB, Dinarello CA (1992). The natural interleukin-1 receptor antagonist in term and preterm parturition. Am. J. Obstet. Gynecol.

[b101] Schellenberg JC (1995). The effect of oxytocin receptor blockade on parturition in guinea pigs. J. Clin. Invest.

[b102] Schlegel A, Volonte D, Engelman JA, Galbiati F, Mehta P, Zhang XL (1998). Crowded little caves: structure and function of caveolae. Cell. Signal.

[b103] Schousboe I, Rasmussen MS (1995). Synchronized inhibition of the phospholipid mediated autoactivation of factor XII in plasma by beta 2-glycoprotein I and anti-beta 2-glycoprotein I. Thromb. Haemost.

[b104] Schutzer WE, Reed JF, Mader SL (2005). Decline in caveolin-1 expression and scaffolding of G protein receptor kinase-2 with age in Fischer 344 aortic vascular smooth muscle. Am. J. Physiol. Heart Circ. Physiol.

[b105] Scott JE, Grigsby PL, Hirst JJ, Jenkin G (2001). Inhibition of prostaglandin synthesis and its effect on uterine activity during established premature labor in sheep. J. Soc. Gynecol. Investig.

[b106] Sengupta P (2013). The laboratory rat: relating its age with human's. Int. J. Prev. Med.

[b107] Sennstrom MB, Ekman G, Westergren-Thorsson G, Malmstrom A, Bystrom B, Endresen U (2000). Human cervical ripening an inflammatory process mediated by cytokines. Mol. Hum. Reprod.

[b108] Shaul PW, Anderson RG (1998). Role of plasmalemmal caveolae in signal transduction. Am. J. Physiol.

[b109] Shiratori I, Ogasawara K, Saito T, Lanier LL, Arase H (2004). Activation of natural killer cells and dendritic cells upon recognition of a novel CD99-like ligand by paired immunoglobulin-like type 2 receptor. J. Exp. Med.

[b110] Simon DB, Karet FE, Hamdan JM, DiPietro A, Sanjad SA, Lifton RP (1996). Bartter's syndrome, hypokalaemic alkalosis with hypercalciuria, is caused by mutations in the Na-K-2Cl cotransporter NKCC2. Nat. Genet.

[b111] Singh H, Endo Y, Nie G (2011). Decidual HtrA3 negatively regulates trophoblast invasion during human placentation. Hum. Reprod.

[b112] Smith RD, Babiychuk EB, Noble K, Draeger A, Wray S (2005). Increased cholesterol decreases uterine activity: functional effects of cholesterol alteration in pregnant rat myometrium. Am. J. Physiol. Cell Physiol.

[b113] Smith GC, Cordeaux Y, White IR, Pasupathy D, Missfelder-Lobos H, Pell JP (2008). The effect of delaying childbirth on primary cesarean section rates. PLoS Med.

[b114] Somara S, Gilmont RR, Martens JR, Bitar KN (2007). Ectopic expression of caveolin-1 restores physiological contractile response of aged colonic smooth muscle. Am. J. Physiol. Gastrointest. Liver Physiol.

[b115] Stepan H, Leo C, Purz S, Höckel M, Horn LC (2005). Placental localization and expression of the cell death factors BNip3 and Nix in preeclampsia, intrauterine growth retardation and HELLP syndrome. Eur. J. Obstet. Gynecol. Reprod. Biol.

[b116] Sugino N, Karube-Harada A, Taketani T, Sakata A, Nakamura Y (2004). Withdrawal of ovarian steroids stimulates prostaglandin F2alpha production through nuclear factor-kappaB activation via oxygen radicals in human endometrial stromal cells: potential relevance to menstruation. J. Reprod. Dev.

[b117] Sykes L, MacIntyre DA, Yap XJ, Teoh TG, Bennett PR (2012). The Th1:th2 dichotomy of pregnancy and preterm labour. Mediators Inflamm.

[b118] Tadagavadi RK, Wang W, Ramesh G (2010). Netrin-1 regulates Th1/Th2/Th17 cytokine production and inflammation through UNC5B receptor and protects kidney against ischemia-reperfusion injury. J. Immunol.

[b119] Tato CM, Joyce-Shaikh B, Banerjee A, Chen Y, Sathe M, Ewald SE (2012). The myeloid receptor PILR*β* mediates the balance of inflammatory responses through regulation of IL-27 production. PLoS ONE.

[b120] Telfer JF, Itoh H, Thomson AJ, Norman JE, Nakao K, Campa JS (2001). Activity and expression of soluble and particulate guanylate cyclases in myometrium from nonpregnant and pregnant women: down-regulation of soluble guanylate cyclase at term. J. Clin. Endocrinol. Metab.

[b121] Temma-Asano K, Tskitishvili E, Kanagawa T, Tomimatsu T, Tsutsui T, Kimura T (2011). Effects of 4-hydroxy-2-nonenal, a major lipid peroxidation-derived aldehyde, and N-acetylcysteine on the cyclooxygenase-2 expression in human uterine myometrium. Gynecol. Obstet. Invest.

[b122] Thomson AJ, Telfer JF, Young A, Campbell S, Stewart CJ, Cameron IT (1999). Leukocytes infiltrate the myometrium during human parturition: further evidence that labour is an inflammatory process. Hum. Reprod.

[b123] Toescu V, Nuttall SL, Martin U, Nightingale P, Kendall MJ, Brydon P (2004). Changes in plasma lipids and markers of oxidative stress in normal pregnancy and pregnancies complicated by diabetes. Clin. Sci. (Lond.).

[b125] Vassalli P (1992). The pathophysiology of tumour necrosis factors. Ann. Rev. Immunol.

[b126] Ventura SJ, Abma JC, Mosher WD, Henshaw SK (2009). Estimated pregnancy rates for the United States, 1990–2005: an update. Natl. Vital Stat. Rep.

[b127] Wang HQ, Takebayashi K, Tsuchida K, Nishimura M, Noda Y (2003). Follistatin-related gene (FLRG) expression in human endometrium: sex steroid hormones regulate the expression of FLRG in cultured human endometrial stromal cells. J. Clin. Endocrinol. Metab.

[b128] Wathes DC, Guldenaar SE, Swann RW, Webb R, Porter DG, Pickering BT (1986). A combined radioimmunoassay and immunocytochemical study of ovarian oxytocin production during the periovulatory period in the ewe. J. Reprod. Fertil.

[b129] Wathes DC, Abayasekara DR, Aitken RJ (2007). Polyunsaturated fatty acids in male and female reproduction. Biol. Reprod.

[b130] Willecke K, Eiberger J, Degen J, Eckardt D, Romualdi A, Guldenagel M (2002). Structural and functional diversity of connexin genes in the mouse and human genome. Biol. Chem.

[b131] Winkler M, Fischer DC, Ruck P, Horny HP, Kemp B, Rath W (1998). Cytokine concentrations and expression and adhesion molecules in the lower uterine segment during parturition at term: relation to cervical dilatation and duration of labour. Z. Gebrtschilfe Neonatol.

[b132] Yahata T, Shao W, Endoh H, Hur J, Coser KR, Sun H (2001). Selective coactivation of estrogen-dependent transcription by CITED1 CBP/p300-binding protein. Genes Dev.

[b133] Yellon SM, Mackler AM, Kirby MA (2003). The role of leukocyte traffic and activation in parturition. J. Soc. Gynecol. Investig.

[b134] Young A, Thomson AJ, Ledingham M, Jordan F, Greer IA, Norman JE (2002). Immunolocalization of pro-inflmmatory cytokines in myometrium cervix and fetal membranes during human parturition at term. Biol. Reprod.

[b135] Yuan W, López Bernal A (2007). Cyclic AMP signalling pathways in the regulation of uterine relaxation. BMC Preg. Childbirth.

[b136] van Zante A, Gauguet JM, Bistrup A, Tsay D, von Andrian UH, Rosen SD (2003). Lymphocyte-HEV interactions in lymph nodes of a sulfotransferase-deficient mouse. J. Exp. Med.

[b137] Zhang J, Bricker L, Wray S, Quenby S (2007). Poor uterine contractility in obese women. BJOG.

[b138] Zhao B, Koon D, Curtis AL, Soper J, Bethin KE (2007). Identification of 9 uterine genes that are regulated during mouse pregnancy and exhibit abnormal levels in the cyclooxygenase-1 knockout mouse. Reprod. Biol. Endocrinol.

[b139] Zhu Z, He X, Johnson C, Stoops J, Eaker AE, Stoffer DS (2007). PI3K is negatively regulated by PIK3IP1, a novel p110 interacting protein. Bioch. Biophys. Res. Commun.

[b140] Zoubina EV, Smith PG (2000). Axonal degeneration and regeneration in rat uterus during the estrous cycle. Auton. Neurosci.

[b141] Zuo J, Lei ZM, Rao CV, Pietrantoni M, Cook VD (1994). Differential cyclooxygenase-1 and -2 gene expression in human myometria from preterm and term deliveries. J. Clin. Endocrinol. Metab.

