# Supplementary material for: Maternal age effects on myometrial expression of contractile proteins, uterine gene expression, and contractile activity during labor in the rat
Source: Physiol Rep. 2015 Apr 15;3(4):e12305. doi: 10.14814/phy2.12305 (PMC4425948; doi:10.14814/phy2.12305)
Supplement: Supplementary file 1 — Table S1. Oligonucleotide primer sequence for quantitative real-time PCR assays. [file phy20003-e12305-sd1.docx]

|  |  |  |  |
| --- | --- | --- | --- |
| **Supplementary Table 1**. Oligonucleotide primer sequence for quantitative real-time PCR assays. | | | |
|  |  |  |  |
| Gene | Primer Sequence | GenBank ascession number | Entrez Gene |
|  |  |  |  |
| *Afp* | Fwd ATGCCAGGACAATATGGAAGAAG | NM_012493.2 | 24177 |
|  | Rev TGGTAGAGATCACAACTTTGTTTGG |  |  |
| *Apob* | Fwd TCTTGCCACAGCTGATCGAA | NM_019287.2 | 54225 |
|  | Rev CCGGCTGTCCACACTGAAT |  |  |
| *Apoh* | Fwd TCCTGTTTGTGCTCGGATCA | NM_001009626.1 | 287774 |
|  | Rev GTATTCCTTAAGGGCTGCAAACTT |  |  |
| *Prl* | Fwd AAAGATAATTAGCCAGGCCTATCCT | NM_012629.1 | 24683 |
|  | Rev CCAAGTCTTTGGATTCTTCATCAA |  |  |
| *Ceacam11* | Fwd TGCTCACAGTCTCCCTTTTAACC | NM_001025404.1 | 292668 |
|  | Rev GCACTGCTTCAATGGTGACTTG |  |  |
| *Apom* | Fwd ACCATTCGCACGAAAAACG | NM_019373.2 | 55939 |
|  | Rev CCGTGTTTCCTTTCCCTTCA |  |  |
| *Ttr* | Fwd CCGTTTGCCTCTGGGAAGA | NM_012681.2 | 24856 |
|  | Rev CCCCTTCCGTGAACTTCTCA |  |  |
| *Nrk* | Fwd CGTACAGGAAATGAGGTCCAAGA | NM_001191797.1 | 315907 |
|  | Rev TCACAGACACTTGGAGAAAATTTCA |  |  |
| *Pramef12* | Fwd GCCGAGAGCCTTGAATGC | XM_006225597.2 | 691157 |
|  | Rev GCCAAGCTGCAGCAAAGC |  |  |
| *Slc12a1* | Fwd TGATGAACAATTTTCAGGTCATGA | XM_008762144.1 | 25065 |
|  | Rev AAGACAGTGTTGCCGAAAAGATC |  |  |
| *Scgb1a1* | Fwd GCAGGAACCCAGCTGAAGAG | NM_013051.1 | 25575 |
|  | Rev TGTTAGGATCTTCTCCGTGAGCTT |  |  |
| *Fxyd3* | Fwd CCCTGAAGATAAAGATAGCCCTTTC | NM_172317.2 | 116831 |
|  | Rev CAGAGAATCCCTGCACAGATGA |  |  |
| *Gabrp* | Fwd CTGCGCCTTGCTCAGTACAC | NM_031029.1 | 81658 |
|  | Rev CAACCGCGTATAGTTTCCTGTCT |  |  |
| *Clic6* | Fwd CCCCCGAGGTACCCTAAGC | NM_176078.2 | 304081 |
|  | Rev TAAACGCTGAGAATTTGGCAAA |  |  |
| *Serpina3m* | Fwd CCATGGTGCTGGTGAACTACCT | NM_001270982.1 | 24795 |
|  | Rev ACAGACCTCTTCTCATCCACGTAGA |  |  |
| *Cxcl6* | Fwd CCGGTCCTGCTCGTCATT | NM_022214.1 | 60665 |
|  | Rev AAGGAGCAGCTTGAGCTCGAT |  |  |
| *Krt85* | Fwd CCAGGATGTGGAGTTACCAGAAA | NM_001008811.1 | 407762 |
|  | Rev CAGCAGCGGTTGCCAGTT |  |  |
| *Ctse* | Fwd GAGGAGTGACCCCAGTGTTTG | NM_012938.1 | 25424 |
|  | Rev CCTTGTGGGTCACTGCTCAA |  |  |
| *Tmprss11g* | Fwd TGACAATTACAAAAACCCCAAACTG | NM_001008554.1 | 289546 |
|  | Rev CTGCGTAGTTCTCGTGAATGATG |  |  |
| *Cd79a* | Fwd AAAGCGTTCCTGTGGCACTT | XM_001060872.5 | 295176 |
|  | Rev GGTGCCTTCCCCCATGTC |  |  |
