# Supplementary material for: Maternal age effects on myometrial expression of contractile proteins, uterine gene expression, and contractile activity during labor in the rat
Source: Physiol Rep. 2015 Apr 15;3(4):e12305. doi: 10.14814/phy2.12305 (PMC4425948; doi:10.14814/phy2.12305)
Supplement: Supplementary file 2 — Table S2. The 129 genes that were significantly different between the older and younger rats. [file phy20003-e12305-sd2.docx]

| **Fold Change**  **Supplementary table 2.** The 129 genes that were significantly different between the older and younger rats. | |  | **Gene name** |  | **Fold Change** | |  | | **Gene name** | |
| --- | --- | --- | --- | --- | --- | --- | --- | --- | --- | --- |
| 5.12 |  | Scgb1a1 |  | | 1.54 |  | RGD1559566 | |  |  |
| 3.01 |  | Fxyd3 |  | | 1.53 |  | Krt75 | |  |  |
| 2.63 |  | Gabrp |  | | 1.52 |  | Ephb2 | |  |  |
| 2.17 |  | Serpina3n |  | | 1.52 |  | Grpr | |  |  |
| 2.16 |  | Clic6 |  | | 1.51 |  | Cxcl14 | |  |  |
| 2.15 |  | Cxcl5 |  | | 1.50 |  | Serpinb5 | |  |  |
| 2.07 |  | Tmprss11g |  | | -1.50 |  | Ctsk | |  |  |
| 2.06 |  | Ctse |  | | -1.51 |  | Wfdc1 | |  |  |
| 2.01 |  | Krt85\|Krt83 |  | | -1.51 |  | Limch1 | |  |  |
| 1.96 |  | Cd79a |  | | -1.52 |  | Cpxm1 | |  |  |
| 1.94 |  | Noxa1 |  | | -1.52 |  | Bnip3\|Bnip3-ps1 | |  |  |
| 1.92 |  | Sctr |  | | -1.52 |  | Col15a1 | |  |  |
| 1.88 |  | Fam3d |  | | -1.52 |  | Metrnl | |  |  |
| 1.87 |  | Lrrc26 |  | | -1.52 |  | Gpx3 | |  |  |
| 1.85 |  | RT1-CE16 |  | | -1.52 |  | Flvcr2 | |  |  |
| 1.84 |  | Ptgs2 |  | | -1.53 |  | Ddit3 | |  |  |
| 1.83 |  | LOC100365794\|LOC688829 |  | | -1.53 |  | LOC302495 | |  |  |
| 1.81 |  | RT1-Bb\|LOC688090 |  | | -1.53 |  | Ccl21 | |  |  |
| 1.79 |  | Dio2 |  | | -1.53 |  | Bmp6 | |  |  |
| 1.79 |  | RGD1566006 |  | | -1.54 |  | LOC100360642 | |  |  |
| 1.75 |  | Fam134b\|Zfp622 |  | | -1.55 |  | Cmtm5 | |  |  |
| 1.75 |  | Inmt |  | | -1.55 |  | Tfpi | |  |  |
| 1.75 |  | Foxa1 |  | | -1.55 |  | Unc5b | |  |  |
| 1.75 |  | LOC100362279\|LOC100364658 |  | | -1.55 |  | Gpnmb | |  |  |
| 1.72 |  | Dlgap1 |  | | -1.55 |  | Htra3 | |  |  |
| 1.71 |  | Grid2 |  | | -1.56 |  | Zim1 | |  |  |
| 1.70 |  | Chst4 |  | | -1.56 |  | Col8a1 | |  |  |
| 1.69 |  | S100vp |  | | -1.56 |  | LOC679690 | |  |  |
| 1.67 |  | Capn13 |  | | -1.56 |  | Pla2g15 | |  |  |
| 1.65 |  | RGD1560556 |  | | -1.57 |  | Gsta4 | |  |  |
| 1.64 |  | Ppp1r1b |  | | -1.57 |  | Pparg | |  |  |
| 1.63 |  | Ptprz1 |  | | -1.57 |  | Nppa | |  |  |
| 1.63 |  | RGD1564463 |  | | -1.58 |  | Gucy1b3 | |  |  |
| 1.63 |  | Slc4a11 |  | | -1.59 |  | Npbwr1 | |  |  |
| 1.62 |  | Cxcl2 |  | | -1.59 |  | Chodl | |  |  |
| 1.59 |  | RGD1310209 |  | | -1.59 |  | Cdkn1c | |  |  |
| 1.59 |  | Adam28 |  | | -1.60 |  | C1qtnf1 | |  |  |
| 1.58 |  | Ano7 |  | | -1.60 |  | Tdo2 | |  |  |
| 1.58 |  | LOC688684 |  | | -1.60 |  | Tmem140 | |  |  |
| 1.57 |  | Nrg1 |  | | -1.61 |  | Adamts15 | |  |  |
| 1.56 |  | Il1rn |  | | -1.61 |  | Cp | |  |  |
| 1.56 |  | Cd300e |  | | -1.61 |  | Gng13 | |  |  |
| 1.56 |  | Slpi |  | | -1.62 |  | Oxt | |  |  |
| 1.56 |  | RGD1565709 |  | | -1.63 |  | LOC100360601\|LOC100364922 | |  |  |
| 1.55 |  | RGD1566270 |  | | -1.65 |  | RGD1564534 | |  |  |
| 1.54 |  | Far2 |  | | -1.65 |  | Hgd | |  |  |

| **Fold Change** |  | **Gene name** |  | **Fold Change** |  | **Gene name** |
| --- | --- | --- | --- | --- | --- | --- |
| -1.66 |  | Slc7a2 |  | -2.20 |  | Kcnk2 |
| -1.67 |  | Fcgrt |  | -2.24 |  | Nat8l |
| -1.67 |  | Tnfrsf9 |  | -2.26 |  | Spinlw1 |
| -1.69 |  | Cyp11a1 |  | -2.28 |  | Chrdl1 |
| -1.69 |  | Figf |  | -2.37 |  | Gcgr |
| -1.69 |  | Apon |  | -2.38 |  | LOC100363005 |
| -1.70 |  | Gadd45a |  | -2.4 |  | Fnd3c2 |
| -1.72 |  | Olr1280 |  | -2.4 |  | Chi3l1 |
| -1.72 |  | Pmel |  | -2.4 |  | Sema6d |
| -1.74 |  | Fcrlb |  | -2.5 |  | Gzmc |
| -1.75 |  | S100b |  | -2.5 |  | Prl6a1 |
| -1.75 |  | Fndc1 |  | -2.5 |  | Tmem37 |
| -1.76 |  | Tnn |  | -2.6 |  | Abp1 |
| -1.76 |  | Plxdc1 |  | -2.7 |  | Prf1 |
| -1.78 |  | Neu2 |  | -2.7 |  | Prl7b1 |
| -1.79 |  | Adam12 |  | -2.8 |  | Olr1 |
| -1.80 |  | Foxo4 |  | -2.9 |  | Gzmb |
| -1.80 |  | C1qtnf5 |  | -2.9 |  | Serpine2 |
| -1.84 |  | Lum |  | -3.0 |  | Mmp3 |
| -1.84 |  | C1qtnf6 |  | -3.0 |  | LOC305103 |
| -1.88 |  | Sfmbt2 |  | -3.1 |  | Taf7l |
| -1.89 |  | Tmem178 |  | -3.3 |  | Slc12a1 |
| -1.90 |  | Slc6a2 |  | -3.3 |  | Apoc2 |
| -1.90 |  | Cited1 |  | -3.6 |  | LOC100365336\|LOC681066 |
| -1.91 |  | Anpep |  | -3.7 |  | Igf2 |
| -1.91 |  | Clic5 |  | -3.7 |  | Pramef12 |
| -1.91 |  | Npr3 |  | -3.8 |  | Prl5a2 |
| -1.92 |  | Fstl3 |  | -3.8 |  | H19\|Mir675 |
| -1.92 |  | Hsd17b2 |  | -3.8 |  | Nppb |
| -1.93 |  | Plau |  | -4.0 |  | Nrk |
| -1.93 |  | Gzmf |  | -4.0 |  | RGD1564657 |
| -1.95 |  | RGD1564162 |  | -4.2 |  | Apom |
| -1.95 |  | Ccr1l1 |  | -4.2 |  | Prl2a1 |
| -1.95 |  | Lrp2 |  | -4.3 |  | Ttr |
| -1.98 |  | Itgbl1 |  | -4.3 |  | Prl4a1 |
| -1.99 |  | Cxcl11 |  | -4.6 |  | Prl7a3 |
| -2.00 |  | Rarres2 |  | -4.8 |  | Ceacam11 |
| -2.01 |  | LOC682330\|LOC100365669 |  | -5.5 |  | Prl8a5 |
| -2.02 |  | Fgf7 |  | -5.7 |  | Prl2c1 |
| -2.05 |  | Cthrc1 |  | -5.8 |  | Prl5a1 |
| -2.07 |  | Mlana |  | -6.9 |  | Apoh |
| -2.08 |  | Sh2d1b2 |  | -8.8 |  | Apob |
| -2.12 |  | Mmp12 |  | -10.0 |  | Afp |
| -2.14 |  | Col6a6 |  |  |  |  |
| -2.16 |  | Mttp |  |  |  |  |
| -2.17 |  | Pik3ip1 |  |  |  |  |

**Supplementary table 2** continued.
