# Supplementary figures and images for: Maternal age effects on myometrial expression of contractile proteins, uterine gene expression, and contractile activity during labor in the rat
Source: Physiol Rep. 2015 Apr 15;3(4):e12305. doi: 10.14814/phy2.12305 (PMC4425948; doi:10.14814/phy2.12305)

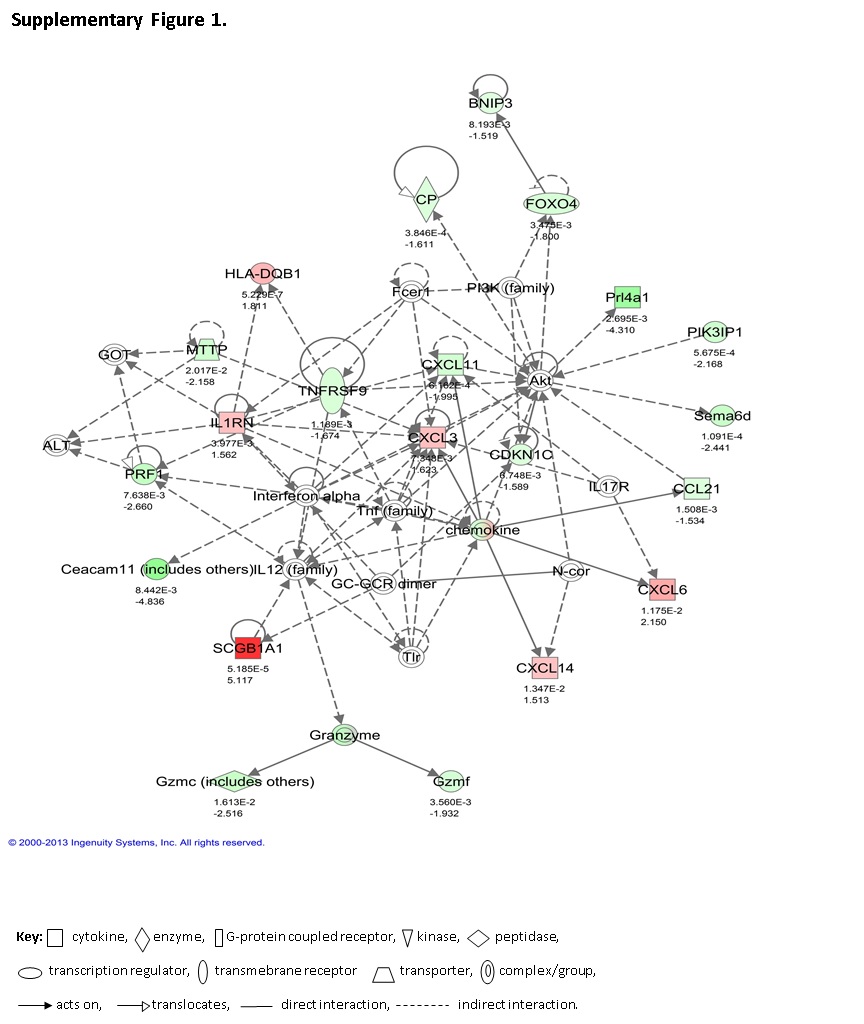

Supplement: Supplementary file 3 — Figure S1. IPA network 1. [file phy20003-e12305-sd3.jpg]

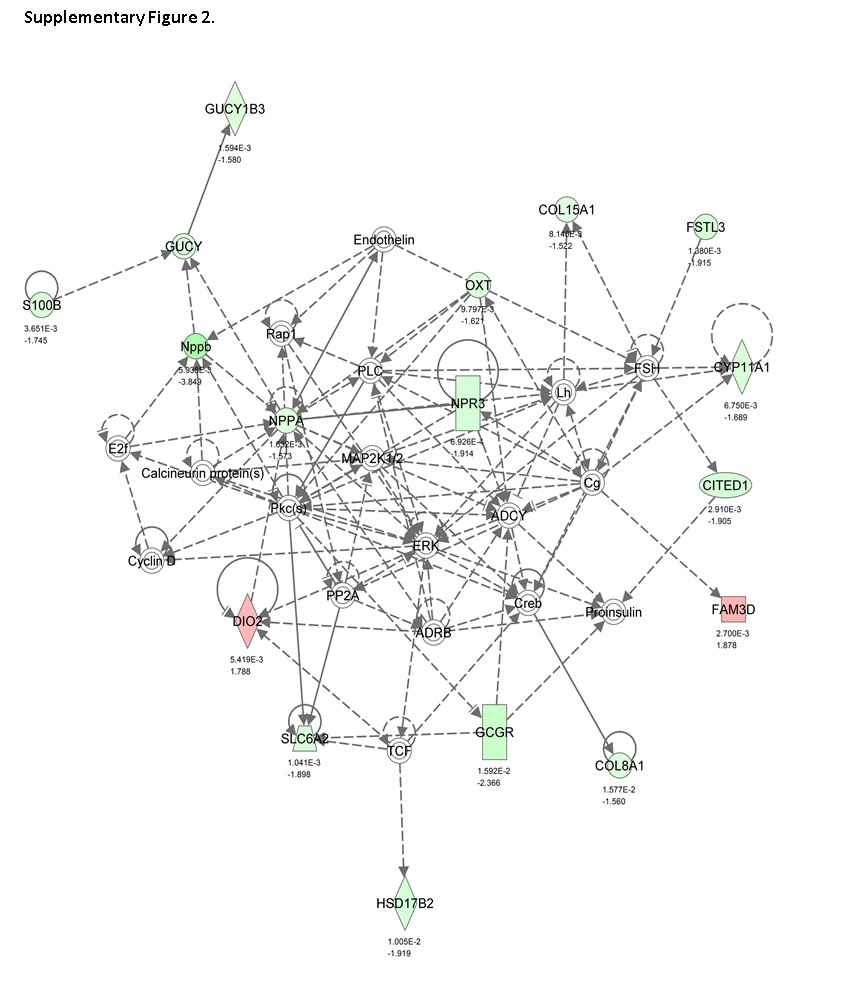

Supplement: Supplementary file 4 — Figure S2. IPA network 2. [file phy20003-e12305-sd4.jpg]

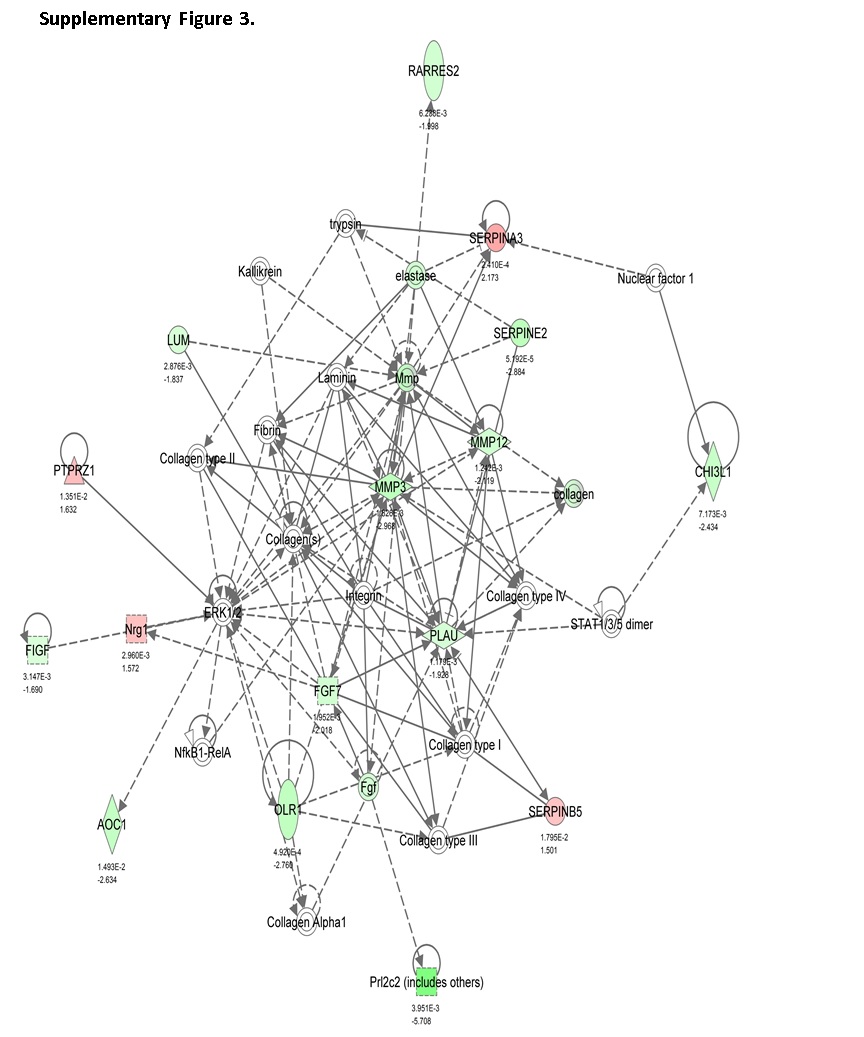

Supplement: Supplementary file 5 — Figure S3. IPA network 3. [file phy20003-e12305-sd5.jpg]

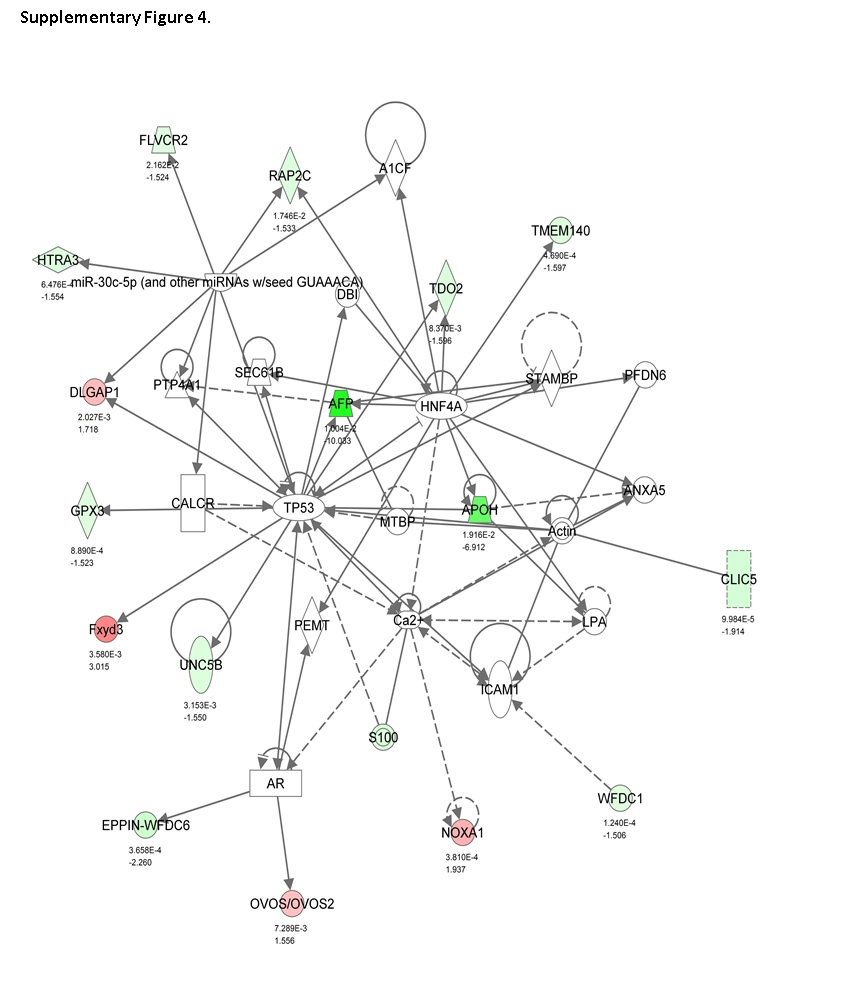

Supplement: Supplementary file 6 — Figure S4. IPA network 4. [file phy20003-e12305-sd6.jpg]

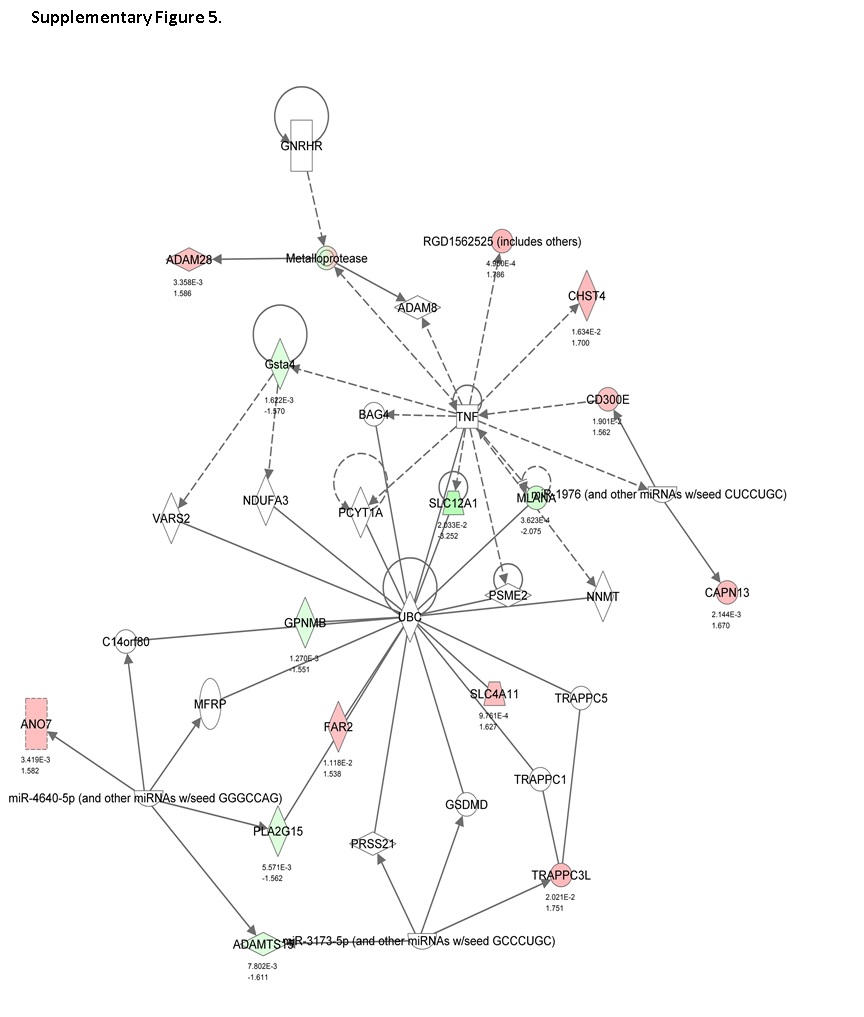

Supplement: Supplementary file 7 — Figure S5. IPA network 5. [file phy20003-e12305-sd7.jpg]
