# Supplementary material for: Maternal age effects on myometrial expression of contractile proteins, uterine gene expression, and contractile activity during labor in the rat
Source: Physiol Rep. 2015 Apr 15;3(4):e12305. doi: 10.14814/phy2.12305 (PMC4425948; doi:10.14814/phy2.12305)
Supplement: Supplementary file 8 [file phy20003-e12305-sd8.docx]

**Supplementary Figure**1 IPA network 1. Differentially regulated genes in uterine horn involved in cell to cell signalling and interaction, Cellular Movement and Immune cell trafficking (Score 40). The network is displayed graphically as nodes (gene/gene products) and edges (the biological relationship between nodes). The node colour intensity indicates the expression of genes: red up-regulated, green down-regulated in the uterine horn of 8 week versus 24 week old labouring rats. The fold changes are indicated under each node. The shapes of the nodes indicate the functional class of the gene product as shown in the key. Solid lines indicate a direct interaction and dotted lines an indirect interaction.

**Supplementary Figure**2**.** IPA network 2. Differentially regulated genes in uterine horn involved in Endocrine System Development and Function, Small Molecule Biochemistry, Cardiovascular System Development and Function (Score 28). The network is displayed graphically as nodes (gene/gene products) and edges (the biological relationship between nodes). The node colour intensity indicates the expression of genes: red up-regulated, green down-regulated in the uterine horn of 8 week versus 24 week old labouring rats. The fold changes are indicated under each node. The shapes of the nodes indicate the functional class of the gene product as shown in the key given in supplementary figure  1. Solid lines indicate a direct interaction and dotted lines an indirect interaction.

**Supplementary Figure**3**.** IPA network 3. Differentially regulated genes in uterine horn involved in Cellular Movement, Cardiovascular System Development and Function, Organismal Development (Score 26). The network is displayed graphically as nodes (gene/gene products) and edges (the biological relationship between nodes). The node colour intensity indicates the expression of genes: red up-regulated, green down-regulated in the uterine horn of 8 week versus 24 week old labouring rats. The fold changes are indicated under each node. The shapes of the nodes indicate the functional class of the gene product as shown in the key given in supplementary figure  1. Solid lines indicate a direct interaction and dotted lines an indirect interaction.

**Supplementary Figure**4**.** IPA network 4. Differentially regulated genes in uterine horn involved in Cell-To-Cell Signalling and Interaction, Haematological System Development and Function, Inflammatory Response (Score 26). The network is displayed graphically as nodes (gene/gene products) and edges (the biological relationship between nodes). The node colour intensity indicates the expression of genes: red up-regulated, green down-regulated in the uterine horn of 8 week versus 24 week old labouring rats. The fold changes are indicated under each node. The shapes of the nodes indicate the functional class of the gene product as shown in the key given in supplementary figure  1. Solid lines indicate a direct interaction and dotted lines an indirect interaction.

**Supplementary Figure 5.** IPA network 5. Differentially regulated genes in uterine horn involved Lipid Metabolism, Small Molecule Biochemistry and Carbohydrate Metabolism (Score 26). The network is displayed graphically as nodes (gene/gene products) and edges (the biological relationship between nodes). The node colour intensity indicates the expression of genes: red up-regulated, green down-regulated in the uterine horn of 8 week versus 24 week old labouring rats. The fold changes are indicated under each node. The shapes of the nodes indicate the functional class of the gene product as shown in the key given in supplementary figure  1. Solid lines indicate a direct interaction and dotted lines an indirect interaction.
